# PTHrP induces STAT5 activation, secretory differentiation and accelerates mammary tumor development

**DOI:** 10.1186/s13058-022-01523-1

**Published:** 2022-04-19

**Authors:** Diego Y. Grinman, Kata Boras-Granic, Farzin M. Takyar, Pamela Dann, Julie R. Hens, Christina Marmol, Jongwon Lee, Jungmin Choi, Lewis A. Chodosh, Martin E. Garcia Sola, John J. Wysolmerski

**Affiliations:** 1 Section of Endocrinology and Metabolism, Department of Internal Medicine, Yale School of Medicine, 300 Cedar Street, TAC S120, Box 208020, New Haven, CT 06520-8020 USA; 2 Research Institute for Endocrine Sciences, Shahid Beheshti University of Medical Sciences, Tehran, Iran; 3 PrimeCare Health, 3924 W Fullerton Ave, Chicago, IL 60647 USA; 4 Brain Korea 21 Plus Project for Biomedical Science, Korea University College of Medicine, Seoul, Korea; 5 Department of Biomedical Sciences, Korea University College of Medicine, Seoul, Korea; 6 Department of Genetics, Yale University School of Medicine, New Haven, CT USA; 7 Department of Cancer Biology, Perlman School of Medicine at the University of Pennsylvania, Philadelphia, PA USA; 8 Departamento de Fisiología y Biología Molecular y Celular, Instituto de Fisiología, Biología Molecular y Neurociencias (IFIByNE), CONICET, Facultad de Ciencias Exactas y Naturales, Universidad de Buenos Aires, Buenos Aires, Argentina

**Keywords:** Lactation, Breast cancer, Milk, Proliferation, Parathyroid hormone-related protein, PyMT, Secretory carcinoma of the breast, PTHLH

## Abstract

**Background:**

Parathyroid hormone-related protein (PTHrP) is required for embryonic breast development and has important functions during lactation, when it is produced by alveolar epithelial cells and secreted into the maternal circulation to mobilize skeletal calcium used for milk production. PTHrP is also produced by breast cancers, and GWAS studies suggest that it influences breast cancer risk. However, the exact functions of PTHrP in breast cancer biology remain unsettled.

**Methods:**

We developed a tetracycline-regulated, MMTV (mouse mammary tumor virus)-driven model of PTHrP overexpression in mammary epithelial cells (Tet-PTHrP mice) and bred these mice with the MMTV-PyMT (polyoma middle tumor-antigen) breast cancer model to analyze the impact of PTHrP overexpression on normal mammary gland biology and in breast cancer progression.

**Results:**

Overexpression of PTHrP in luminal epithelial cells caused alveolar hyperplasia and secretory differentiation of the mammary epithelium with milk production. This was accompanied by activation of Stat5 and increased expression of E74-like factor-5 (Elf5) as well as a delay in post-lactation involution. In MMTV-PyMT mice, overexpression of PTHrP (Tet-PTHrP;PyMT mice) shortened tumor latency and accelerated tumor growth, ultimately reducing overall survival. Tumors overproducing PTHrP also displayed increased expression of nuclear pSTAT5 and Elf5, increased expression of markers of secretory differentiation and milk constituents, and histologically resembled secretory carcinomas of the breast. Overexpression of PTHrP within cells isolated from tumors, but not PTHrP exogenously added to cell culture media, led to activation of STAT5 and milk protein gene expression. In addition, neither ablating the Type 1 PTH/PTHrP receptor (PTH1R) in epithelial cells nor treating Tet-PTHrP;PyMT mice with an anti-PTH1R antibody prevented secretory differentiation or altered tumor latency. These data suggest that PTHrP acts in a cell-autonomous, intracrine manner. Finally, expression of PTHrP in human breast cancers is associated with expression of genes involved in milk production and STAT5 signaling.

**Conclusions:**

Our study suggests that PTHrP promotes pathways leading to secretory differentiation and proliferation in both normal mammary epithelial cells and in breast tumor cells.

**Supplementary Information:**

The online version contains supplementary material available at 10.1186/s13058-022-01523-1.

## Background

Parathyroid hormone-related protein (PTHrP) was originally discovered as a cause of elevated calcium levels in patients with cancer [1–3]. It is evolutionarily related to parathyroid hormone (PTH) and the amino-terminal portions of both proteins are highly homologous, allowing them to bind and activate the same Type 1 PTH/PTHrP receptor (PTH1R) [2, 3]. As a result, when PTHrP is secreted by tumors, it mimics PTH, leading to excessive bone resorption and hypercalcemia. PTHrP also contributes to the development and physiologic functions of a variety of tissues, and it has been shown to affect cell proliferation and cell death in a number of settings [2–5]. While many of its functions are mediated by the PTH1R, PTHrP can also remain within the cell to regulate proliferation, differentiation and survival through an intracrine mode of action requiring the translocation of PTHrP into the nucleus [3, 6–10]. Although nuclear translocation appears to be important for PTHrP biology, details of this signaling pathway remain obscure.

PTHrP and the PTH1R are expressed throughout the life cycle of the mammary gland as well as in breast tumors. Both molecules are required for fetal breast development in mice and humans [11–14]. PTHrP also has important functions during lactation. Its production is greatly upregulated in alveolar epithelial cells, and it is secreted into both milk and the maternal circulation [15–17]. In the maternal circulation, PTHrP acts on bone cells to mobilize calcium from the skeleton that is subsequently used by the mammary gland for milk production. In addition, PTHrP in milk regulates total body calcium accrual in suckling neonates, acting to coordinate maternal and neonatal calcium economy [18].

PTHrP is also produced by breast cancers, contributing both to their growth and to tumor-induced changes in systemic metabolism [5, 15, 19]. When produced by breast cancer cells within the bone microenvironment, PTHrP contributes to osteolytic bone destruction and the expansion of bone metastases [5, 20, 21]. In addition, genome-wide association (GWAS) studies have implicated the *PTHLH* (PTHrP) gene as a breast cancer susceptibility locus [15, 22–24], suggesting that it may contribute to early steps in transformation and/or cancer progression. However, the exact functions of PTHrP in breast cancer biology remain unsettled. Different studies have reported that its expression either correlates with increased or decreased metastases and survival [10, 25–29]. Moreover, studies have variably reported that PTHrP either stimulates or inhibits the proliferation, differentiation and survival of breast cancer cells [3, 5, 10, 21, 30–33]. These contradictory results concerning the role and prognostic value of PTHrP expression in breast cancer underscore the need to better understand how it modulates breast tumor growth and/or breast cancer susceptibility.

In order to examine the effects of PTHrP on mammary tumor development in mice, we developed a tetracycline-regulated, MMTV-driven model of PTHrP overexpression in mammary epithelial cells (MMTV-rtTA;TetO-PTHrP). We found that overexpression of PTHrP in luminal epithelial cells caused alveolar hyperplasia and secretory differentiation of the mammary epithelium enabling virgin mice to produce milk. This phenotype was associated with activation of STAT5, and increased expression of Elf5 (E74-like factor-5), both important regulators of alveolar secretory differentiation [34–37]. Furthermore, overexpression of PTHrP in epithelial cells in MMTV-PyMT mice dramatically promoted the formation of mammary tumors by shortening tumor latency and accelerating tumor growth, ultimately reducing overall survival. Interestingly, tumors overproducing PTHrP expressed markers of secretory differentiation and expressed milk constituents. These data suggest that PTHrP promotes pathways leading to secretory differentiation in both normal mammary epithelial cells and in breast tumor cells.

## Methods

### Animals

We used FVB female mice of various genotypes described below in all experiments. Male mice were not used because the focus of the study was on mammary gland development and breast cancer. All animal experiments were performed in accordance with institutional regulations after protocol review and approval by Yale University’s Institutional Animal Care and Use Committee.

Six different genetically engineered mouse models were used in this study: MMTV-rtTA, MMTV-rtTA;TetO-PTHrP, MMTV-PyMT, MMTV-rtTA;TetO-PTHrP;MMTV-PyMT, MMTV-rtTA;TetO-PTHrP;MMTV-PyMT;MMTV-Cre and, MMTV-rtTA;TetO-PTHrP;MMTV-PyMT;MMTV-Cre;PTH1R^lox/lox^. We used a bi-transgenic, tetracycline-regulated, mouse mammary tumor virus long terminal repeat (MMTV) system to control the timing of PTHrP overexpression. MMTV-rtTA mice from the Chodosh laboratory (University of Pennsylvania) [38] were bred to TetO-PTHrP responder mice generated by the Wysolmerski laboratory [39] to make the double transgenic MMTV-rtTA;TetO-PTHrP (Tet-PTHrP) mice. Although the amino acid sequences of mouse and human PTHrP(1–141) are highly homologous [40] and the two peptides have identical functions, we used the a human PTHrP(1–141) cDNA in creating the TetO-PTHrP mice in order to be able to distinguish transgene-derived mRNA from the endogenous mouse *Pthlh* mRNA.

MMTV-PyMT [41] mice were purchased from Jackson Laboratories on a FVB background and bred into our Tet-PTHrP mice to generate MMTV-rtTA;TetO-PTHrP;MMTV-PyMT (Tet-PTHrP;PyMT) mice. MMTV-Cre [42] (Jackson Laboratories) and PTH1R^fl/fl^ mice (from Henry Kronenberg, Boston, MA) [43] were bred into the MMTV-rtTA;TetO-PTHrP;MMTV-PyMT mice to generate MMTV-rtTA;TetO-PTHrP;MMTV-PyMT;PTH1R^lox/lox^ (Tet-PTHrP;PyMT;PTH1RLox) and MMTV-rtTA;TetO-PTHrP;MMTV-PyMT;MMTV-Cre;PTH1R^lox/lox^ (Tet-PTHrP;PyMT;Cre;PTH1RLox) mice. Doxycycline (Dox) (2 mg/ml; Research Products International, Cat# D43020) was administered in 5% sucrose water and mice could drink ad libitum. Mice were followed weekly for tumors. Once palpable, tumor size was measured weekly with calipers and tumor volume calculated using the formula 0.5 × length × width^2^. Mice were euthanized when tumors reached approximately 1.5 cm in any dimension, or when they appeared unhealthy during the course of the experiment, whichever was earlier.

### Biochemical measurements

Serum calcium concentrations were measured using the Quantichrom Calcium Assay Kit (DICA-500, BioAssay Systems) according to manufacturer's instructions. Plasma PTHrP was measured using an immunoradiometric assay (DSL-8100; Beckman Coulter) in which we substituted a rabbit anti-PTHrP (1–36) antibody generated in our laboratory as capture antibody. This assay has a sensitivity of 0.3 pM. Serum mPRL levels were measured by homologous double-antibody RIAs as previously described [44].

### Whole-mount analysis

Whole-mount analysis was performed on mammary tissue as previously described [45]. Briefly, the no. 4 inguinal mammary glands were removed and mounted on a microscope slide. The tissue was fixed in acid ethanol for 1 h at room temperature, washed in 70% ethanol and then distilled water and incubated in carmine aluminum stain (0.2% carmine, 0.5% aluminum potassium sulfate) overnight at room temperature. After staining, the mammary glands were dehydrated through graded ethanol and cleared in acetone and then toluene before being mounted under glass coverslips using Permount (Fisher Scientific, Cat# SP15-100).

### Histology and immunohistochemistry

Two hours prior to euthanasia, mice were injected with BrdU (Roche) or EdU (50 mg/kg, Invitrogen). Whole mammary glands, tumors and lungs were removed, weighed and fixed for 12 h in 4% paraformaldehyde. After fixation in 4% paraformaldehyde, tissues were transferred to 70% ethanol, embedded in paraffin and cut in 5-μm-thick sections. Pertinent slides were then either stained with hematoxylin and eosin using standard conditions, used for immunohistochemistry, or processed for measuring proliferation using anti-Bromodeoxyuridine-POD, Fab fragment Kit (Roche, Cat# 11585860001) or the Click-iT EdU Cell Proliferation Kit (Invitrogen Cat# C10337). Rates of proliferation were calculated by dividing the number of BrdU- or EdU-positive nuclei by the total number of nuclei. Lungs were processed for histology and pulmonary metastases quantified by examination of 10, H&E-stained sections cut 105 µm apart. All immunohistochemistry included an IgG isotype control, and the primary antibodies we used were against phospho-Stat5 (Cell Signaling, Cat# 9314), β-casein (Santa Cruz Biotechnology, Cat# sc-166530), Elf-5 (Santa Cruz Biotechnology, Cat# sc-9645), NF1B (Sigma, Cat# HPA-0039556), Nkcc1 (gift from Dr. James Turner at National Institutes of Health) and Npt2b (gift from Dr. Jürg Biber at University of Zurich). Staining was detected using Vector Elite ABC kits (Vector Laboratories), Envision Plus (DAKO), or M.O.M. Immunodetection Kit (Vector Laboratories, Cat) and we used 3,3′-diaminobenzidine as a chromogen.

### RNA extraction and real-time RT-PCR

Mammary glands and tumors were homogenized in 1 ml TRIzol (Invitrogen, Cat# 15596018) using an Ultraturrax T25 (Ika Labortechnik) on ice. Lysates were cleared at 13,000 g for 10 min at 4 °C. The RNA was isolated using PureLink RNA columns (Invitrogen, Cat# 12183025) according to the manufacturer’s instructions. Total RNA was quantified using a Nanodrop 1000 spectrophotometer (Thermo Fisher Scientific). For all samples, the ratio of absorbance at 260 nm to absorbance at 280 nm was > 1.8. cDNA was synthesized using 1 μg of total RNA with the High Capacity cDNA Reverse Transcription Kit (Applied Biosystems, Cat# 4368814) according to the manufacturer instructions. Quantitative RT-PCR was performed using the Taqman Fast Universal PCR Master Mix (Applied Biosystems, Cat# 4352042) or Sybr Green PCR Master kit (Applied Biosystems, Cat# 4309155) and a StepOnePlus real-time PCR system (Applied Biosystems). The following TaqMan primer sets were used: hPthlh Hs00174969_m1, mPthlh Mm00436057, PTH1R Mm00441046, Wap Mm00839913_m1, Lalba Mm00495258_m1, Csn1s1 Mm01160593_m1, Csn1s2a Mm00839343_m1, Csn1s2b Mm00839674_m1, Csn2 Mm04207885_m1, Csn3 Mm02581554_m1, Elf5 Mm00468732_m1, Nfib Mm01257777_m1, Gata3 Mm01337570_m1, Hprt1 Mm03024075_m1, Actb (Cat# 4352933E). The following primer pairs for Sybr green were also used: Pymt fwd (5′-ctgctactgcacccagacaa-3′) and Pymt rev (5′-gcaggtaagaggcattctgc-3′), Actb fwd (5′-ccacacccgccaccagttc-3′) and Actb rev (5′-gacccattcccaccatcacacc-3′). Relative mRNA expression was determined using the standard curve method with the StepOne software v2.3 (Applied Biosystems).

### Tissue protein isolation and western blot

Pieces of mammary gland or mammary tumor no more than 0.5 cm × 0.5 cm were lysed in 1 ml of RIPA lysis buffer (10 mM Tris·HCl pH 8, 140 mM NaCl, 1 mM EDTA pH 8, 0.5 mM EGTA pH 8, 1% Triton X-100, 0.1% deoxycholate, and 0.1% SDS) supplemented with a cocktail of protease inhibitors (Thermo Scientific Cat# 78429), 50 mM NaF, and 1 mM Na3VO4 on ice. Samples were then homogenized using an Ultraturrax T25 (Ika Labortechnik). Lysates were centrifugated at 13,000 g for 10 min at 4 °C, and the supernatant was recovered. The samples were quantitated for total protein using the Bradford protein assay (Bio-Rad Cat# 5000001) following the manufacturer’s instructions. A 2 µg/µl protein solution containing sample buffer (Invitrogen Cat# NP0007) plus sample reducing agent (Invitrogen Cat# NP0004) was prepared and 30ug of total protein were loaded into precast, 4% to 12% Bis–Tris acrylamide gels (Thermo Fisher Scientific, Cat# NP0322) in MOPS buffer (Thermo Fisher Scientific, Cat# NP0001) and underwent electrophoresis, after which samples were transferred to nitrocellulose membranes (Bio-Rad, Cat# 1621112). Membranes were treated with blocking buffer (LI-COR Biosciences, Cat# 927-60001) for 1 h at room temperature and then incubated with the primary antibody overnight at 4 °C, followed by a dye conjugated secondary antibody for 1 h at room temperature. Membranes were imaged and analyzed using the Odyssey IR imaging system (LI-COR Biosciences). The primary antibodies used were: anti-PTHrP (Peprotech, Cat# 500-P276), anti-β-casein (Santa Cruz Cat# 166530), anti-Elf5 (Santa Cruz Cat# sc-9645), anti-NF1B (Sigma, Cat# HPA003956), anti-p(Tyr694)Stat5 (Cell Signaling, Cat# #9359), anti-Stat5 (Cell Signaling, Cat# #94205), anti-Npt2b (gift from Dr. Jürg Biber at University of Zurich), anti-β-Actin (Santa Cruz Cat# sc-130656). The secondary antibodies used were anti-mouse (LI-COR, Cat# 926-68022) and anti-Rabbit (LI-COR Biosciences, Cat# 926-32213).

### Tumor cell isolation and culture

Tumor cells were isolated from transgenic mammary tumors as previously described. Briefly, dissected tumors were minced into fragments under sterile conditions and subjected to enzymatic digestion with Collagenase-Type3 (Worthington, Cat#: LS004183) at 2 mg/ml, Dispase (Gibco, Cat#: 17105-041) at 2 mg/ml, Gentamycin (Gibco, Cat#:15710-064) at 50 µg/ml, Amphotericin B (Sigma, Cat#:A2942) at 250 µg/ml, and 5% FBS in DMEM/F12 media for 3 h with intermittent shaking. Following digestion, tumor organoids were pelleted and then treated with NH4Cl (Stem Cell Technologies, Cat # 07800) to lyse RBCs, following which, the pellet was washed three times with PBS. Organoids were then passed through a 70 µm cell strainer, counted and used for transplantation experiments or cultured at a density of 3 × 10^6^ cells/55cm^2^. Proliferation of cultured cells was measured by assessing BrdU incorporation (Cell proliferation ELISA Kit 11647229001; Roche) after addition of Dox (2 µg/ml) or PTHrP (Bachem, Cat# 4017147.0500) to the culture media.

### Tumor cell transplantation

500,000 freshly isolated, sterile tumor cells were suspended in 150 µl of sterile saline and were injected subcutaneously into the fat pad of 8 wild-type, adult FVB mice via a small incision between the fourth nipple and the midline as previously described [46]. Mice were treated with Dox 24 h prior to the injection and were monitored for tumor development. Mice were checked twice a week for tumors and tumor size was measured with calipers every other day. Tumor-bearing animals were euthanized when the tumors reached approximately 1.5 cm in any dimension or when they appeared unhealthy, whichever was earlier.

### Global gene expression profiling

Total RNA was prepared using TRIzol reagent (Invitrogen) from FACS sorted luminal epithelial cells of 4.5 week-old, MMTV-rtTA and Tet-PTHrP mice on Dox from birth, using antibodies against CD24 and CD49f cell surface markers as previously described [47]. Similarly, total RNA was prepared from whole tumor lysates of MMTV-PyMT and Tet-PTHrP;PyMT mice on Dox. The isolated RNA was purified using the RNeasy cleanup kit (Qiagen). RNA was reverse-transcribed and hybridized to Affymetrix Mouse Genome 430 2.0 GeneChip by the Yale Center for Genomic Analysis. Microarray data were analyzed with R version 4.1.2 and Bioconductor 3.14 [48]. Raw data were MAS5 normalized and log_2_ transformed. 20,000 probes with the highest statistical significance were selected as the first working matrix, and then, only genes with fold change of +/− 2 and *p* < 0.01 were considered for further analyses. Differentially expressed genes (DEGs) were analyzed using WikiPathways Pathway Analysis for biological interpretation [49], and significant pathways were based on the Bonferroni adjusted *p* value (padj) < 0.05. Results of the functional analysis were combined and integrated to the expression data with the GOplot package [50]. All statistical analyses and data visualization plots were done with R/Bioconductor packages. GSEA analysis was performed using previously generated set of ∼200 STAT5-dependent and mammary tissue restricted genes [37]. Enrichment score curves and member ranks were generated by the GSEA software package [51]. Volcano plots were constructed from the first selected 20,000 probes matrix with *ggplot2* [52]. Heatmap was generated with *heatmap.2* package.

### Breast cancer single cell RNA seq data download and process

Count matrices from published single cell RNA sequencing (scRNA-seq) datasets were downloaded from the NCBI Gene Expression Omnibus (GSE161529) and then analyzed using Seurat version 4.0 [53]. Seurat objects were created from 15 ER+ , 6 HER2+ and 4 TNBC patient-derived datasets. Cells with > 60,000 counts and the number of unique genes detected in each cell were removed using > 200 and < 7000 as criteria. This is a quality control step, as it is thought that cells with high numbers of counts are more likely to be doublets, while cells with low numbers of counts are thought to be of poor data quality. Data normalization, variable feature detection, feature scaling, and principal component analysis were performed in Seurat using default parameters. Cell clusters were identified using the default Louvain clustering algorithm implemented in Seurat. Default Seurat function settings were used except that clustering resolutions were set to 0.5 and principal component dimensions 1:10 were used for all dimension reduction and integration steps. Epithelial cells were identified using canonical marker genes as described, and normalized counts data were used in all relevant downstream analysis [54]. Cells were divided into two groups depending on their normalized counts of *PTHLH* expression level. *PTHLH* high groups expressed *PTHLH* more than 0, and remaining cells were designated as the *PTHLH* low group. Differential expression between PTHLH high and low groups was conducted using the FindMarker function in Seurat package with MAST option. Pathway enrichment was performed on ranked lists with fGSEA using HALLMARK gene set from MsigDB v7.4 [51, 55]. After removing genes that are not expressed in any cell, protein coding genes only were considered (refer to *biomaRt* package [56]).

### Statistics

Results were expressed as means ± SE of at least 3 independent experiments. Statistical analyses were performed with Prism 9.0 (GraphPad Software) and consisted of one-way ANOVA, followed by Tukey’s multiple comparisons test. Before statistical analysis, Q-Q plot and Shapiro–Wilk test were performed for normality. Homoscedasticity was assessed with Levene’s test. In figures, asterisks mean significant differences between means.

## Results

### Overexpression of PTHrP in luminal epithelial cells causes alveolar hyperplasia

**Fig. 1:**
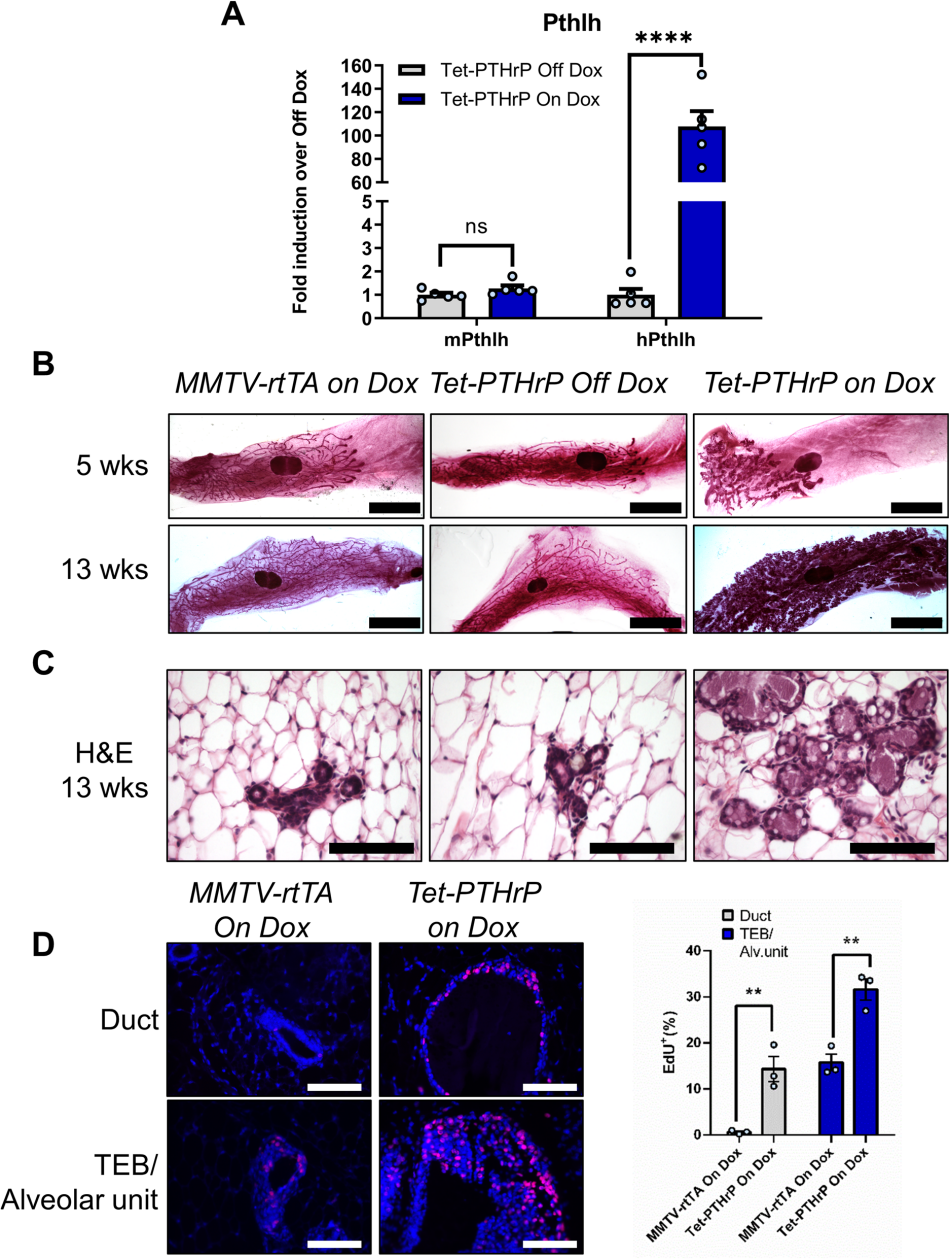
PTHrP overexpression causes alveolar hyperplasia. **A** Relative expression of mouse and human *PTHLH* mRNA in mammary gland lysates. *Hprt1* was used as housekeeping gene. Bars represent mean ± SEM of fold change versus Tet-PTHrP off dox, *n* = 5 mice per group. **B** Whole-mount analysis of carmine-stained number 4 inguinal mammary glands from mice at 5 and 13 weeks. Scale bar 5 mm. **C** H&E stained cross-sections from 13-week-old mice. Scale bar 100 µm. **D** Representative images and quantification of EdU incorporation in sections of mammary glands from mice at 5 weeks of age. Bar graphs represent the percentage of Edu-positive cells over a minimum of 1000 total nuclei (DAPI). *N* = 3 mice per group, ****p < 0.0001 **p < 0.01

We created a tetracycline-regulated model of PTHrP overexpression using a well-described MMTV-rtTA mouse that employs the mouse mammary tumor virus long terminal repeat (MMTV) to drive expression of the reverse tetracycline transactivator (rtTA) in mammary epithelial cells (MECs) [57]. When MMTV-rtTA mice were bred to a mouse containing a tetracycline-responsive, human PTHrP transgene, (TetO-PTHrP mice) [39], the resulting double-transgenic, MMTV-rtTA;TetO-PTHrP (Tet-PTHrP) offspring demonstrated a significant induction of human *PTHLH* mRNA in mammary glands upon treatment with Dox (Fig. 1A). As expected, there was essentially no human *PTHLH* mRNA expressed in mammary glands from Tet-PTHrP mice in the absence of Dox, nor was there induction of the endogenous mouse *Pthlh* gene in response to Dox.

In previous studies, overexpression of PTHrP in myoepithelial cells delayed ductal elongation and this was also the case in Tet-PTHrP mice treated with Dox [39]. As shown in Fig. 1B, at 5 weeks of age, the ducts in Tet-PTHrP virgin mice off Dox had grown past the central lymph node and displayed a dichotomous branching pattern typical of the virgin mammary gland. By contrast, in Tet-PTHrP mice treated with Dox, the ducts were foreshortened but hyperplastic in appearance. Ducts in MMTV-rtTA mice on Dox were the same as those in Tet-PTHrP mice off Dox, demonstrating that the effects observed were due to PTHrP and not to either Dox or rtTA. By 13 weeks of age, the ducts in all genotypes had advanced to the borders of the fat pad but the glands of Dox-treated Tet-PTHrP mice displayed obvious alveolar hyperplasia on whole mount, reminiscent of normal glands in mid to late pregnancy. In fact, histologic examination of mammary glands from the Dox-treated Tet-PTHrP mice demonstrated multiple clusters of MECs forming alveolar structures (Fig. 1C; Additional file 1: Fig. S1A). Since there was no apparent structural or developmental difference between MMTV-rtTA mice on Dox and Tet-PTHrP mice off Dox, nor leakiness of human PTHrP expression in the latter, subsequent experiments used either of these genotypes interchangeably as controls.

Given the increased numbers of epithelial cells in the glands from Tet-PTHrP mice treated with Dox, we assessed rates of epithelial cell proliferation by measuring EdU incorporation. As shown in Fig. 1D, in response to PTHrP overexpression, there was a clear increase in EdU incorporation in MECs in both ducts and terminal end buds (TEBs), which looked abnormal in appearance and were often found together in close proximity with the hyperplastic alveolar units (Additional file 1: Fig. S1A). These data demonstrate that induction of PTHrP expression in MECs leads to alveolar hyperplasia.

### Overexpression of PTHrP activates secretory differentiation of mammary epithelial cells

Overexpression of PTHrP was accompanied by distension of the hyperplastic alveolar and ductal lumens with what appeared to be secretory material. In addition, large lipid droplets were apparent in both the cells and the luminal space (Fig. 1C). These features suggested milk production, and milk-like fluid was evident upon gross inspection of the intact mammary glands of Tet-PTHrP mice treated with Dox (Additional file 1: Fig. S1B).

**Fig. 2:**
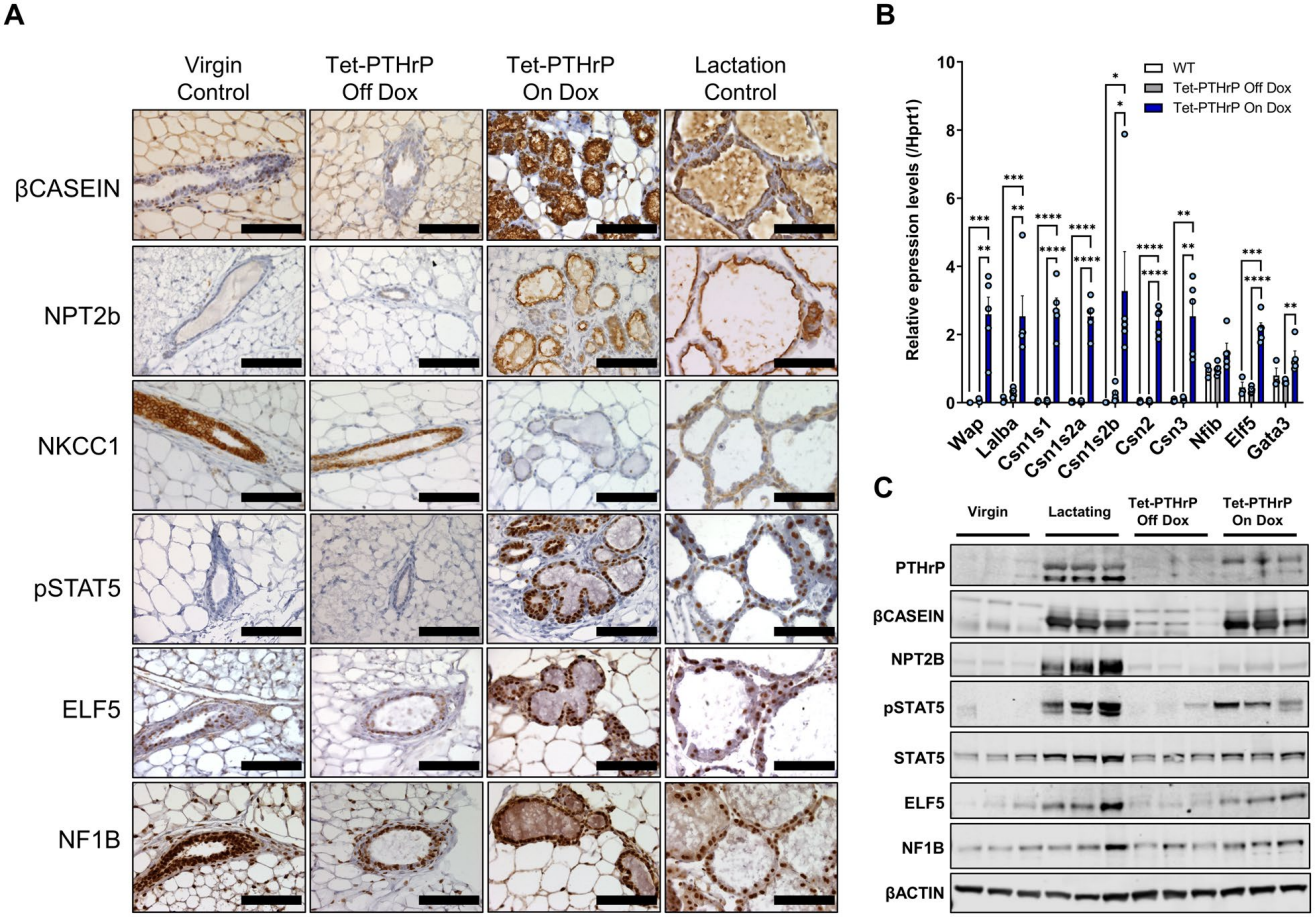
PTHrP overexpression induces the expression of milk proteins and markers of secretory differentiation. **A** Immunohistochemical staining of mammary gland sections of 8–12-week-old mice. Representative images from each group are shown. *N* = 3, Scale bar 100 µm. **B** QPCR analysis for the expression of milk protein and transcription factor genes from whole mammary glands of 8–12-week-old mice. *Hprt1* was used as housekeeping gene. **C** Western blot analysis of protein lysates from mammary glands of 8–12-week-old mice. Samples from three different mice per group were run with β-Actin as the loading control. Bars represent mean ± SEM, *n* = 3 per group, ****p < 0.0001, ***p < 0.001, **p < 0.01, *p < 0.05

In order to confirm that MECs underwent secretory differentiation in response to PTHrP, we assayed differentiation markers typically expressed by MECs during lactation [34, 58]. As shown in Fig. 2A, Dox treatment induced the expression of β-casein and the sodium-phosphate transporter 2b (NPT2b) as measured by immunohistochemistry, while suppressing expression of the sodium–potassium-chloride co-transporter (NKCC1) in MECs of virgin Tet-PTHrP mice. These changes were identical to normal lactating controls but were absent in normal virgin controls and in virgin Tet-PTHrP mice in the absence of Dox. We also assayed the expression of a series of milk-protein genes by QPCR (Fig. 2B). Overexpression of PTHrP caused the induction of whey acidic protein (*Wap*), alpha lactalbumin (*Lalba*) and multiple casein genes, none of which were expressed in the glands from virgin controls or in Tet-PTHrP mice off Dox. PTHrP overexpression led to a significant increase in PTHrP protein levels and, as expected from the gene expression data and the immunostaining, an increase in β-casein and NPT2b protein expression in whole mammary gland lysates as assessed by immunoblot (Fig. 2C; Additional file 1: Fig. S1C). Importantly, the MMTV-driven PTHrP overexpression did not alter circulating PTHrP levels or prolactin (Prl) levels, suggesting that the secretory differentiation of the MECs is likely to be a local effect of PTHrP rather than an indirect effect of circulating Prl (Additional file 1: Fig. S1D).

Next, we examined whether alveolar hyperplasia and/or secretory differentiation in MECs required ongoing exposure to PTHrP. Female Tet-PTHrP mice were placed on Dox at 8 weeks of age for 4 weeks and then were either euthanized immediately or followed for an additional 6 weeks off Dox before being euthanized. Additional controls included similar nulliparous, Tet-PTHrP mice treated with Dox for 10 continuous weeks before euthanasia. As expected, PTHrP expression for 4 weeks or 10 weeks in adult females triggered alveolar hyperplasia, activated the expression of β-casein and NPT2b, and suppressed NKCC1 expression (Additional file 2: Fig. S2). After the withdrawal of Dox for 6 weeks, the alveolar hyperplasia had almost completely resolved histologically, NPT2b staining was no longer observed, and NKCC1 became evident. However, MECs continued to express some β-casein, albeit principally within the lumen of the ducts and at lower levels than in MECs from glands exposed to ongoing PTHrP overexpression. These results suggest that alveolar hyperplasia and the mature secretory phenotype requires ongoing exposure to PTHrP but that some changes in cell differentiation or cell fate may persist after transient exposure to PTHrP.

### Overexpression of PTHrP induces changes in gene expression similar to lactation

Secretory differentiation of MECs during normal pregnancy and lactation requires changes in gene expression driven by several pioneering transcription factors including pSTAT5, Elf 5 and Nuclear factor 1B (NF1B) [34, 37, 59, 60]. As seen in Fig. 2A, immunohistochemistry demonstrated that Dox treatment of virgin Tet-PTHrP mice induced the expression of pSTAT5 within MEC nuclei, mimicking the pattern typically seen during lactation. There was also a clear increase in pSTAT5 in immunoblot analyses of mammary glands taken from Tet-PTHrP mice on Dox that was not present in Tet-PTHrP mice off Dox (Fig. 2C; Additional file 1: Fig. S1). While nuclear staining for Elf5 was evident in MECs from virgin controls and from Tet-PTHrP mice off Dox, the staining intensity appeared increased in MECs from virgin Tet-PTHrP mice on Dox and in lactating control mice (Fig. 2A). This impression was confirmed by an increase in *Elf5* mRNA as assessed by QPCR (Fig. 2B) as well as increased Elf5 protein levels as measured by immunoblot (Fig. 2C; Additional file 1: Fig. S1C). Finally, expression of NF1B as assessed by immunohistochemistry, QPCR and immunoblot was not clearly different in lactating mammary glands or in mammary glands from Tet-PTHrP mice on Dox as compared with glands from either control Tet-PTHrP mice off Dox or virgin mice. Continued full expression of these transcription factors required the ongoing presence of PTHrP, because withdrawal of PTHrP expression resulted in substantial reduction, although not complete elimination of the immunostaining for pSTAT5 and Elf5. As before, expression of NF1B was not affected by PTHrP expression (Additional file 2: Fig. S2).

**Fig. 3:**
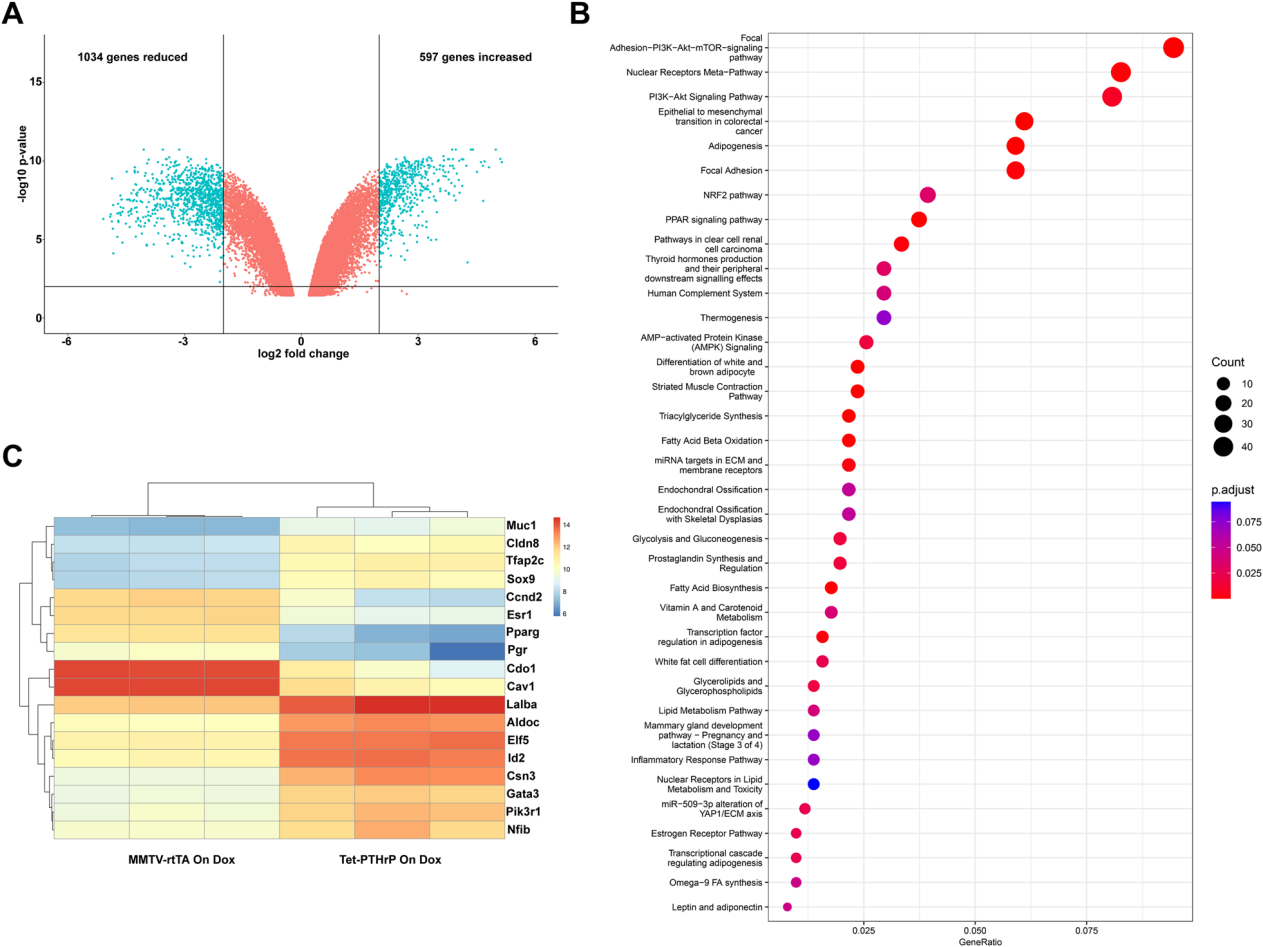
PTHrP induces changes in genes involved in secretory differentiation in luminal cells. Global mRNA profiling was performed in FACS sorted luminal mammary epithelial cells isolated from 4.5-week-old MMTV-rtTA and Tet-PTHrP mice on Dox from birth. **A** Volcano plot shows the log2 fold change and variance for all transcripts in Tet-PTHrP cells relative to controls. Lines illustrate twofold changes and a padj of 0.01. Differentially expressed transcripts are highlighted in light blue and the number of genes increased or decreased is indicated. **B** Heatmap showing relative expression change of representative genes involved in mammary gland secretory differentiation. **C** Pathway analysis on differentially expressed genes. Node size represents gene count; node color represents padj

We next performed an analysis of overall gene expression using oligonucleotide-based microarrays. We compared mRNA expression patterns from luminal MECs isolated from Tet-PTHrP mice on Dox to that of luminal MECs isolated from MMTV-rtTA control mice on Dox. Using a log fold change (LFC) cutoff of 2 and an adjusted p value of 0.01, we found 1631 genes differentially expressed (597 increased and 1034 reduced) as a result of PTHrP expression (Fig. 3A). Pathway analysis demonstrated that the differentially expressed transcripts comprised key pathways important for MEC secretory differentiation, including PI3K/Akt signaling, fatty acid biosynthesis, triglyceride biosynthesis and the mammary gland transition from pregnancy to lactation (although this did not quite reach statistical significance, padj = 0.07), among others (Fig. 3B). A more detailed analysis of the genes involved in alveolar cell differentiation revealed an increase in the levels of *Elf5, Nf1b, Gata3, Sox9, Csn3, Tfap2c, PiK3r1, Lalba, Cldn8* and Muc1 as well as a downregulation of *Esr1, Pgr, Cav1, Cdo1* and *Ccnd2* transcripts, all changes consistent with the activation of a secretory program in luminal cells similar to lactation [61–63] (Fig. 3C). Taken together, these results demonstrate that increased levels of PTHrP are sufficient to induce the expression of lactation-associated transcription factors and patterns of gene expression in the absence of a prior pregnancy.

### Overexpression of PTHrP delays involution

**Fig. 4:**
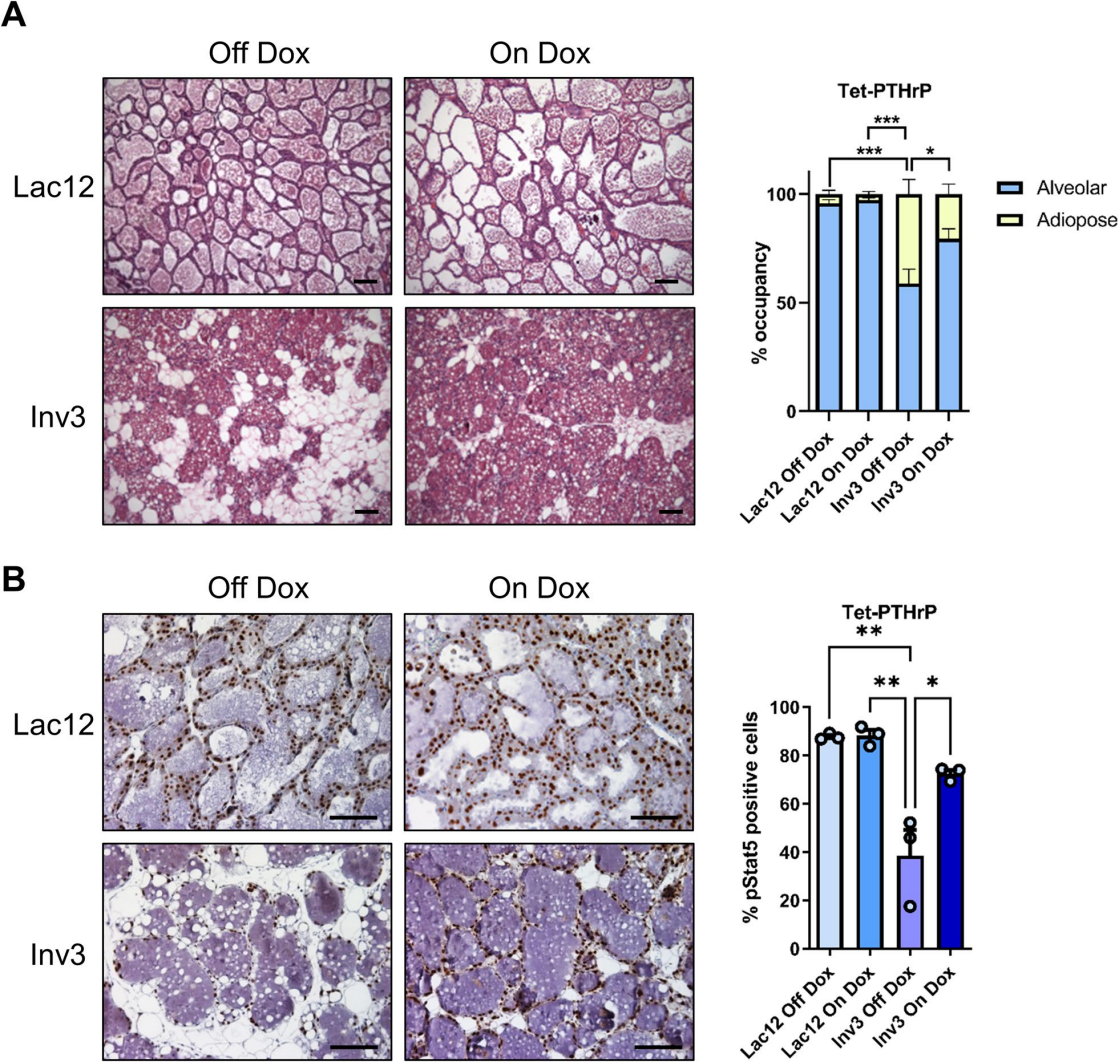
PTHrP maintains the expression of activated STAT5 and delays involution. H&E (**A**) and immunohistochemical analysis (**B**) of mammary glands from lactation day 12 (Lac12) and involution day 3 (Inv3) glands from Tet-PTHrP mice on Dox versus off Dox as controls. **A** Bars represent mean ± SEM of the area occupied by adipose and alveolar tissue in 10 random fields. **B** Bars represent mean ± SEM of the percentage of pStat5 positive cells in 5 random fields (average of 2200 cells per gland). Representative images from each group are shown. *N* = 3, Scale bar 100 µm

STAT5 is a survival factor for mammary epithelial cells [64], and its activation is downregulated at the onset of post-lactational involution [65, 66]. If transgenic expression of PTHrP caused persistent STAT5 activation, it might impair the involution response. To test this hypothesis, female Tet-PTHrP mice were treated with Dox or vehicle from mating and through lactation—and were subsequently sacrificed at day 12 of lactation (Lac12) or after 12 days of lactation followed by 3 days of forced weaning (Inv3). Mammary glands were then examined histologically. As shown in Fig. 4A, the sections from lactating mice off Dox and on Dox had identical histology consistent with normal lactation. In addition, the pups suckled and grew normally. Therefore, PTHrP overexpression in these mice does not impair normal lactation. However, PTHrP overexpression did delay involution. There was a clear difference in the relative proportion of epithelial tissue vs. adipose tissue that can be seen in the representative histology at 3 days of involution and that is quantified by the histomorphometric analysis shown in the bar graphs to the right (Fig. 4A). These changes in tissue remodeling were accompanied by changes in pSTAT5 staining as shown in the immunohistochemistry panels in Fig. 4B. When we calculated the percentage of epithelial cells staining positive for pSTAT5, the control glands had the expected decrease at 3 days after weaning as compared to mid-lactation. However, PTHrP-overexpressing glands (Tet-PTHrP on dox) showed a statistically significant retention of pSTAT5 staining, consistent with the delayed involution seen histologically. These data demonstrate that PTHrP overexpression maintains the expression of activated STAT5, which is known to promote the inappropriate survival of mammary epithelial cells after weaning, delaying the involution of the alveolar structures in the gland [67].

### Overexpression of PTHrP accelerates tumor formation in MMTV-PyMT mice

**Fig. 5:**
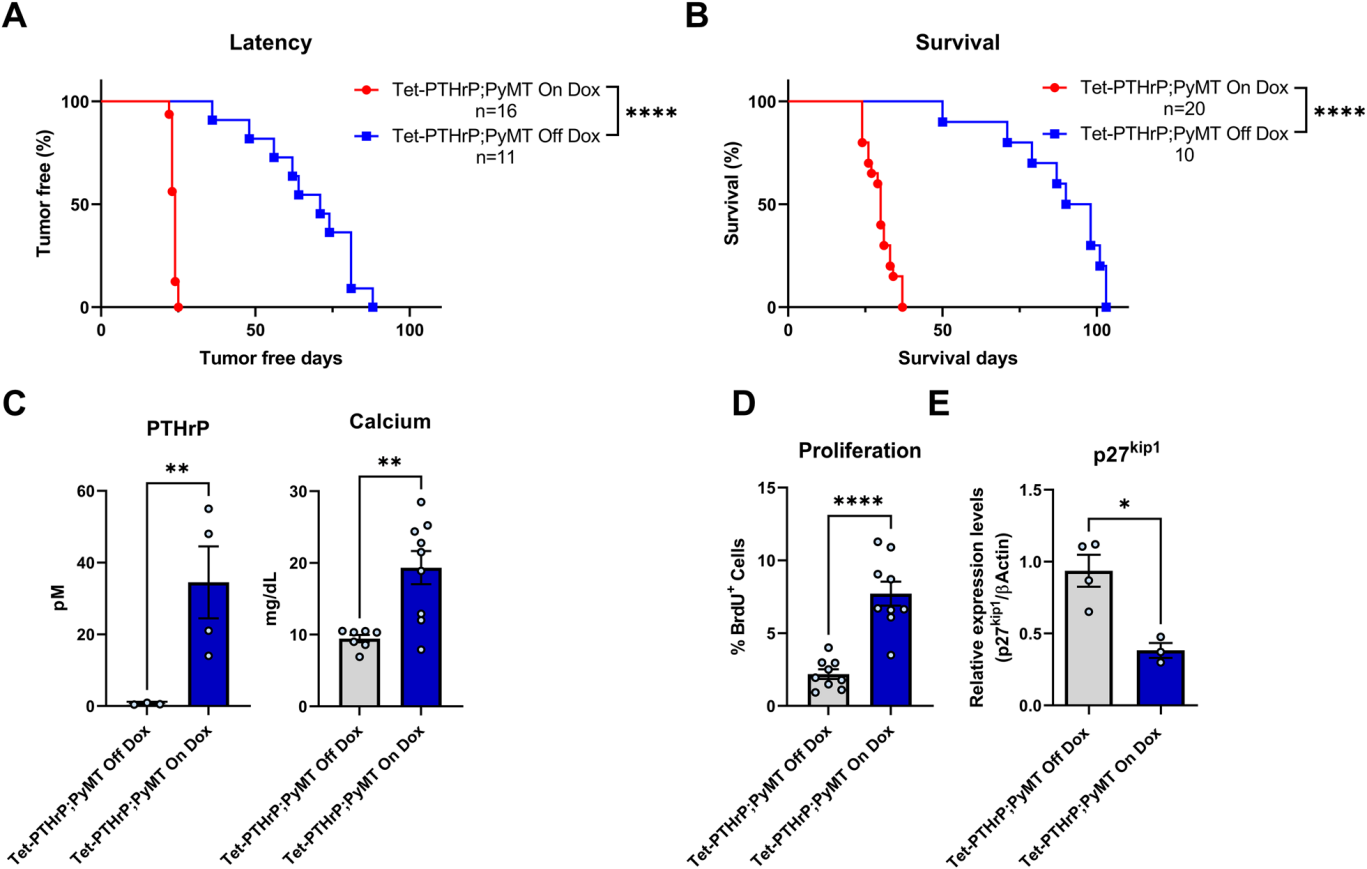
Overexpression of PTHrP accelerates tumor formation in MMTV-PyMT mice. Kaplan–Meier analysis of **A** tumor onset and **B** survival in Tet-PTHrP;PyMT mice on Dox (red) versus Tet-PTHrP;PyMT mice off Dox (blue). **C** Circulating levels of plasma PTHrP and serum calcium concentration. **D** Quantitation of BrdU incorporation in tumor sections. Results are expressed as the percentage of BrdU-positive cells over a minimum of 1000 total cells. **E** Expression levels of *p27kip1* mRNA relative to *β-actin* mRNA in tumors. Bars represent mean ± SEM, a minimum of *n* = 3 per group, ****p < 0.0001, **p < 0.01, *p < 0.05

We observed a cohort of 6 Tet-PTHrP mice on Dox for over a year (median 417 days) to determine whether the alveolar hyperplasia associated with PTHrP overexpression would result in the formation of mammary tumors. Only 1 mouse developed a tumor at 354 days, suggesting that PTHrP, itself, was not an efficient or dominant oncoprotein. However, in order to determine whether PTHrP overexpression might influence tumor formation caused by an established oncogene, we bred the MMTV-PyMT transgene onto Tet-PTHrP mice to generate MMTV-rtTA;TetO-PTHrP;MMTV-PyMT (Tet-PTHrP;PyMT) mice [68]. Continuous PTHrP overexpression from birth led to a dramatic acceleration of tumor formation (Fig. 5A). Microscopic tumors developed in Tet-PTHrP;PyMT mice as early as 5–10 days of age (Additional file 3: Fig. S3A) and 100% of the mice had palpable masses in all mammary glands by just over 20 days of age (median latency of 24 days) (Fig. 5A). In contrast, control Tet-PTHrP;PyMT mice maintained off Dox developed tumors in only some glands between 40 and 90 days with a median latency of 71 days (Fig. 5A). PTHrP overexpression also dramatically shortened survival (Fig. 5B). When treated with Dox, Tet-PTHrP;PyMT mice became systemically ill, developed high circulating PTHrP and calcium levels (Fig. 5C), and died before 40 days of age (median survival of 30 days). In contrast, control Tet-PTHrP;PyMT mice off Dox appeared generally healthy, had normal PTHrP and calcium levels, but were euthanized due to tumor size between 50 and 100 days of age (median survival of 94 days). Importantly, overexpression of PTHrP did not increase the expression of the MMTV-PyMT transgene in Tet-PTHrP;PyMT tumors nor in cells isolated from them (Additional file 3: Fig. S3B), demonstrating that acceleration of tumorigenesis was caused by PTHrP and not by increased PyMT expression.

BrdU staining demonstrated that PTHrP increased cell proliferation in mammary tumors from Tet-PTHrP;PyMT mice on Dox (Fig. 5D). Previous work has shown that PTHrP can regulate G1-S cell-cycle progression in vascular smooth muscle and breast cancer cells by modulating expression of the cell-cycle inhibitor, p27kip1 [69, 70]. Therefore, we examined p27kip1 levels in tumors harvested from Tet-PTHrP;PyMT mice on or off Dox and found that increasing PTHrP production decreased p27kip1 levels in mammary tumors (Fig. 5E).

### PTHrP overexpression leads to secretory differentiation of MMTV-PyMT tumor cells

**Fig. 6:**
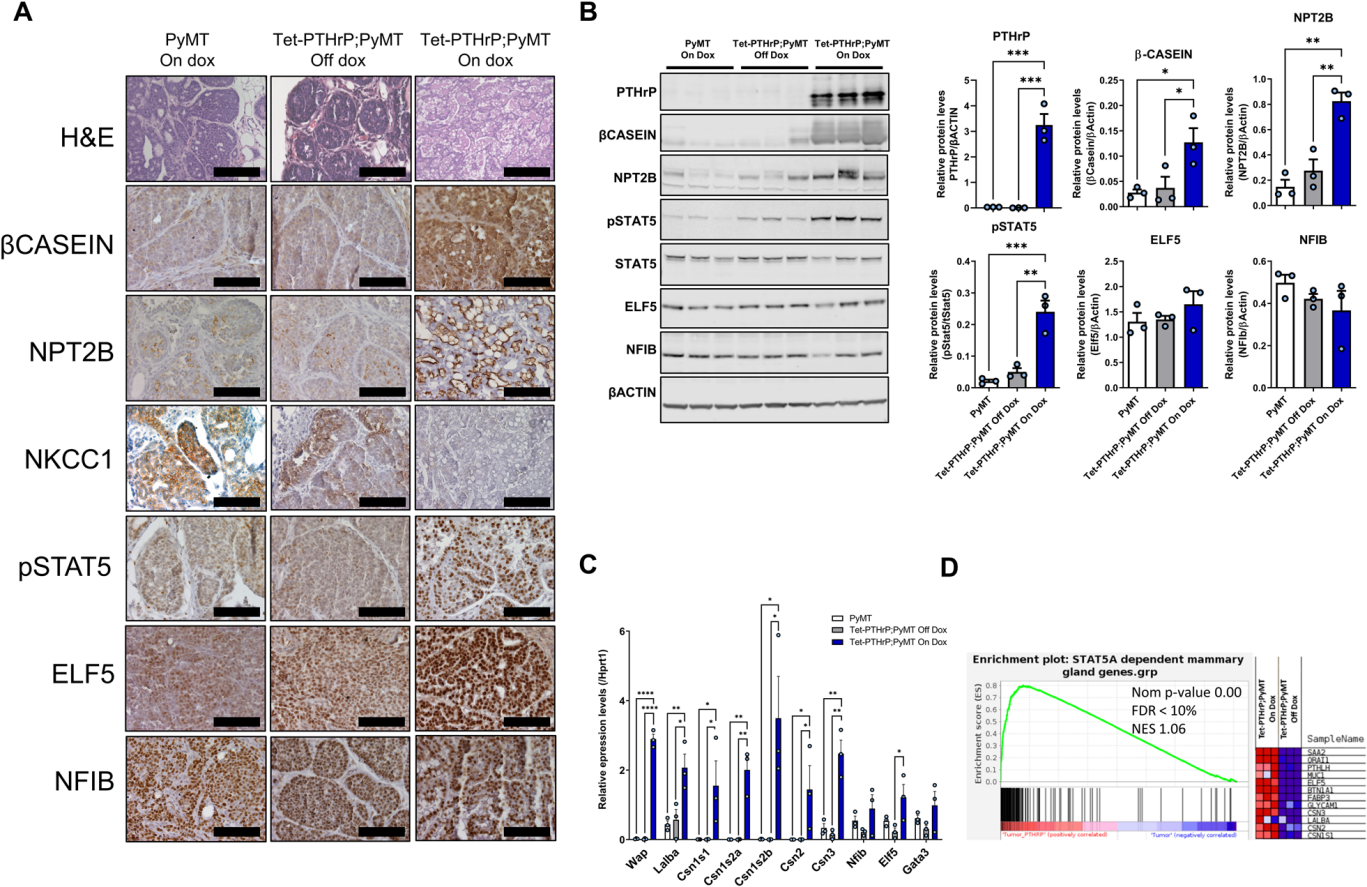
PTHrP overexpression causes secretory differentiation and Stat5 activation in PyMT tumors. **A** H&E and immunohistochemical analysis of tumors from Tet-PTHrP;PyMT mice on Dox and controls. Representative images from each group are shown. *N* = 3. **B** Western Blots on protein lysates from whole tumors. Left shows Samples from three different mice per group were run with β-Actin as the loading control. Right shows the densitometric quantification of western blots. **C** QPCR analysis for the indicated genes of RNA isolated from whole tumors. *Hprt1* was used as housekeeping gene. *N* = 3. **D** Left shows the custom GSEA for STAT5-dependent mammary gland genes comparing Tet-PTHrP;PyMT vs PyMT mice on dox. Nom *p*-value, normalized *p* value; FDR, false discovery rate; NES, normalized enrichment score. Right, heatmap depicting relative expression change of representative STAT5-dependent genes. Bars represent mean ± SEM, ****p < 0.0001, ***p < 0.001, **p < 0.01, *p < 0.05

Histologically, the tumors from Tet-PTHrP;PyMT mice on Dox displayed a papillary phenotype and prominent intracellular lipid droplets as well as secretory material in extracellular “lumens” between fronds of tumor cells (Fig. 6A; Additional file 4: Fig. S4A). In addition, tumor dissection often revealed the presence of viscous white fluid resembling milk (Additional file 4: Fig. S4B). These changes were reminiscent of the secretory differentiation seen in MECs of non-tumor bearing mice overexpressing PTHrP (Fig. 2). Therefore, we performed immunohistochemistry to examine the same mammary differentiation markers in tumor cells (Fig. 6A). Interestingly, tumors from control MMTV-PyMT mice on Dox demonstrated low levels of immunostaining for β-casein, NPT2b and pSTAT5, although expression of all three of these markers was significantly upregulated in tumors from Tet-PTHrP;PyMT mice on Dox. In addition, NKCC1 expression was downregulated by PTHrP expression. Nuclear staining for Elf5 appeared more prominent in tumors overexpressing PTHrP but nuclear staining for NF1B appeared unchanged. Western blot analyses from whole tumor lysates demonstrated similar findings. Tumors from Tet-PTHrP;PyMT mice on Dox displayed significantly higher levels of pSTAT5, β-casein and NPT2b than tumors from either MMTV-PyMT mice on Dox or from Tet-PTHrP;PyMT mice off Dox (Fig. 6B). Elf5 and NF1B levels in tumors taken from Tet-PTHrP;PyMT mice on Dox were not statistically significantly different from controls. QPCR from whole tumors revealed a significant elevation of *Wap*, *Lalba* and the different casein mRNA levels in response to PTHrP overexpression. There was also a small increase in *Elf5* gene expression but no change in *Nf1b* or *Gata3* gene expression. Overall, these changes mirrored the activation of secretory differentiation induced by PTHrP in normal MECs without the PyMT oncogene.

Given the apparent increased level of differentiation of the cells, we also examined whether tumors in Tet-PTHrP;PyMT mice on Dox continued to demonstrate malignant behavior. First we examined lungs from these mice for metastases (Additional file 4: Fig. S4C) and found that 6 out of 10 Tet-PTHrP;PyMT mice treated with Dox developed lung lesions. Among those, we counted an average of 6.17 lung metastasis per mouse, documenting that tumor cells retained the ability to disseminate and metastasize to distant sites. Importantly, the tumor cells that metastasized to the lungs continued to stain positive for pStat5 in the metastatic lesions, suggesting that continuous activation of this factor by PTHrP does not affect the metastatic process (Additional file 4: Fig. S4D). We also transplanted isolated tumor cells from Tet-PTHrP;PyMT mice into mammary fat pads of WT animals. In the presence of Dox, 75% of the 8 mice receiving these cells developed tumors that secreted PTHrP into the circulation producing significant hypercalcemia (Additional file 4: Fig. S4E). These data demonstrate that PTHrP overexpression did not extinguish the tumor-propagating potential of the cells. Therefore, while PTHrP triggered a program of secretory differentiation in PyMT tumor cells, it did so without reversing their transformed state.

### Mammary tumors overexpressing PTHrP activate gene signatures that overlap with STAT5 signaling and lactation

We reasoned that the development of secretory alveolar hyperplasia in the mammary glands of the Tet-PTHrP mice and the formation of secretory adenocarcinomas in Tet-PTHrP;PyMT mice are likely to be a consequence of the activation of common STAT5-dependent pathways. To test this hypothesis, we performed a second microarray using RNA isolated from tumors of Tet-PTHrP;PyMT mice on or off Dox. As before, we used a LFC cutoff of 2 and an adjusted p value of 0.01 and identified a total of 921 differentially expressed genes (686 reduced and 235 increased) in response to PTHrP overexpression (Additional file 5: Fig. S5A). Pathway analysis demonstrated that the differentially expressed transcripts could be grouped into pathways involved in adipocyte differentiation, fatty acid metabolism and the mammary gland transition from pregnancy to lactation, among others (Additional file 5: Fig. S5B). We then asked specifically whether mammary epithelial cell, STAT5-dependent genes were activated in tumors overexpressing PTHrP by comparing the differentially expressed genes from PTHrP-overexpressing tumors to a previously validated set of ~ 200 STAT5-dependent genes specific to mammary tissue [37]. Gene set enrichment analysis demonstrated that overexpression of PTHrP led to a significant enrichment of Stat5-dependent mRNAs in PyMT-derived tumors (Fig. 6D). To illustrate the induction of STAT5-dependent genes, the accompanying heatmap shows relative changes in the expression of 12 selected STAT5 target genes that are normally induced during lactation and are also induced by PTHrP expression in tumors. The complete list of the genes from the set and their relative change in expression in response to PTHrP overexpression is shown in Additional file 5: Fig. S5C.

**Fig. 7:**
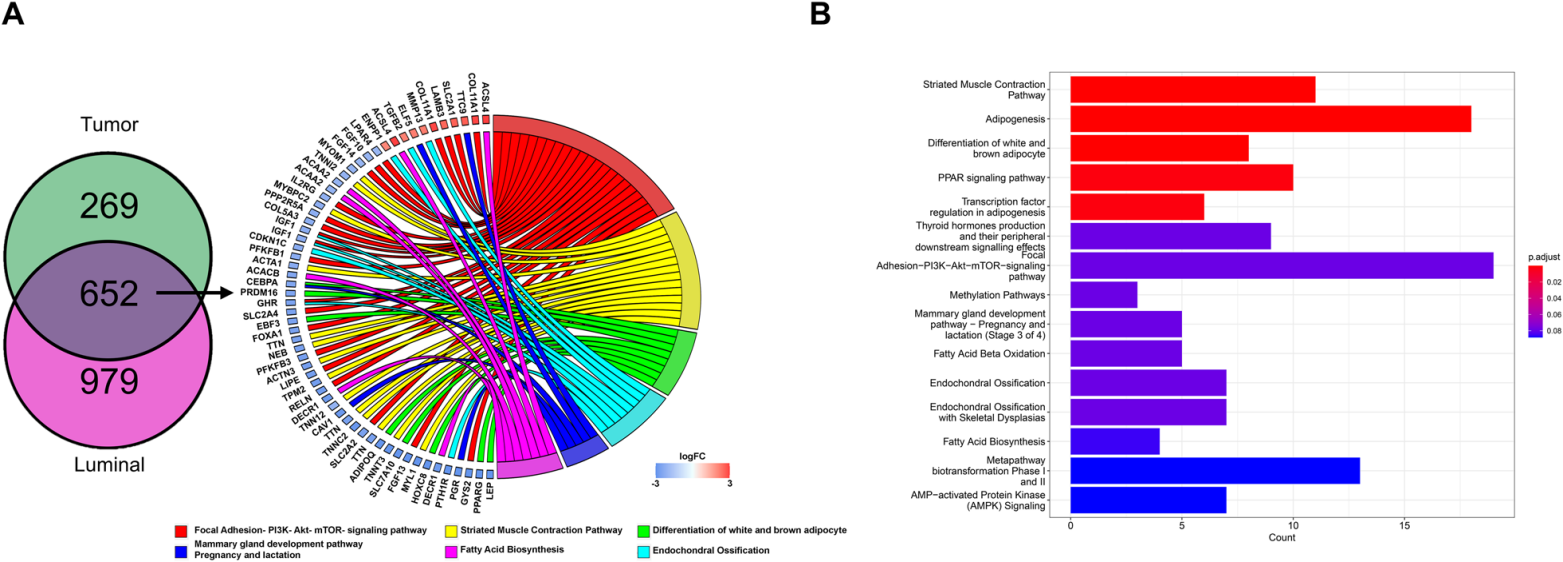
Identification of overlapping genes in mammary epithelial cells and mammary tumors overexpressing PTHrP. **A** (Left) Venn diagram indicating overlap between differentially expressed genes in PTHrP-overexpressing MECs (purple) and tumors (green). (Right) Chord plot illustrating a detailed relationship between the log_2_-fold change (log_2_FC) of overlapped DEGs (left semicircle) and their enriched selected biological pathways. **B** Pathways analysis on differentially expressed overlapped genes. Bar length represents gene count; bar color represents padj

Given the similarities in the secretory phenotypes induced by PTHrP in normal MECs and in PyMT tumors, we directly compared differentially expressed genes (DEGs) in PTHrP-overexpressing tumors from Tet-PTHrP;PyMT mice with DEGs in PTHrP-overexpressing MECs from Tet-PTHrP mice (Fig. 7). There were 921 DEGs in PTHrP-overexpressing tumors and 1,631 DEGs in PTHrP overexpressing luminal MECs. Comparing these sets of genes documented a substantial overlap with a shared group of 652 genes (524 reduced and 128 increased) that were differentially expressed in both settings. Analysis of the genes in the overlap showed expression of genes involved in mammary gland development skewed toward lactation, as indicated by upregulation of *Elf5* and *Ttc* and downregulation of *Cebpα*, *Cav-1* and progesterone receptor (*Pgr*) (Fig. 7B). Stimulation of ELF5 and downregulation of Cav-1, PGR, and changes in the PI3K/Akt pathway are all consistent with an increase in STAT5 signaling [34, 37, 60–63, 71, 72]. Overall, these results are consistent with the idea that PTHrP overexpression leads to STAT5 activation and secretory differentiation in both normal MECs as well as in PyMT-induced mammary tumors.

### Activation of Stat5 in tumor cells by PTHrP is cell autonomous and independent of PTH1R

**Fig. 8:**
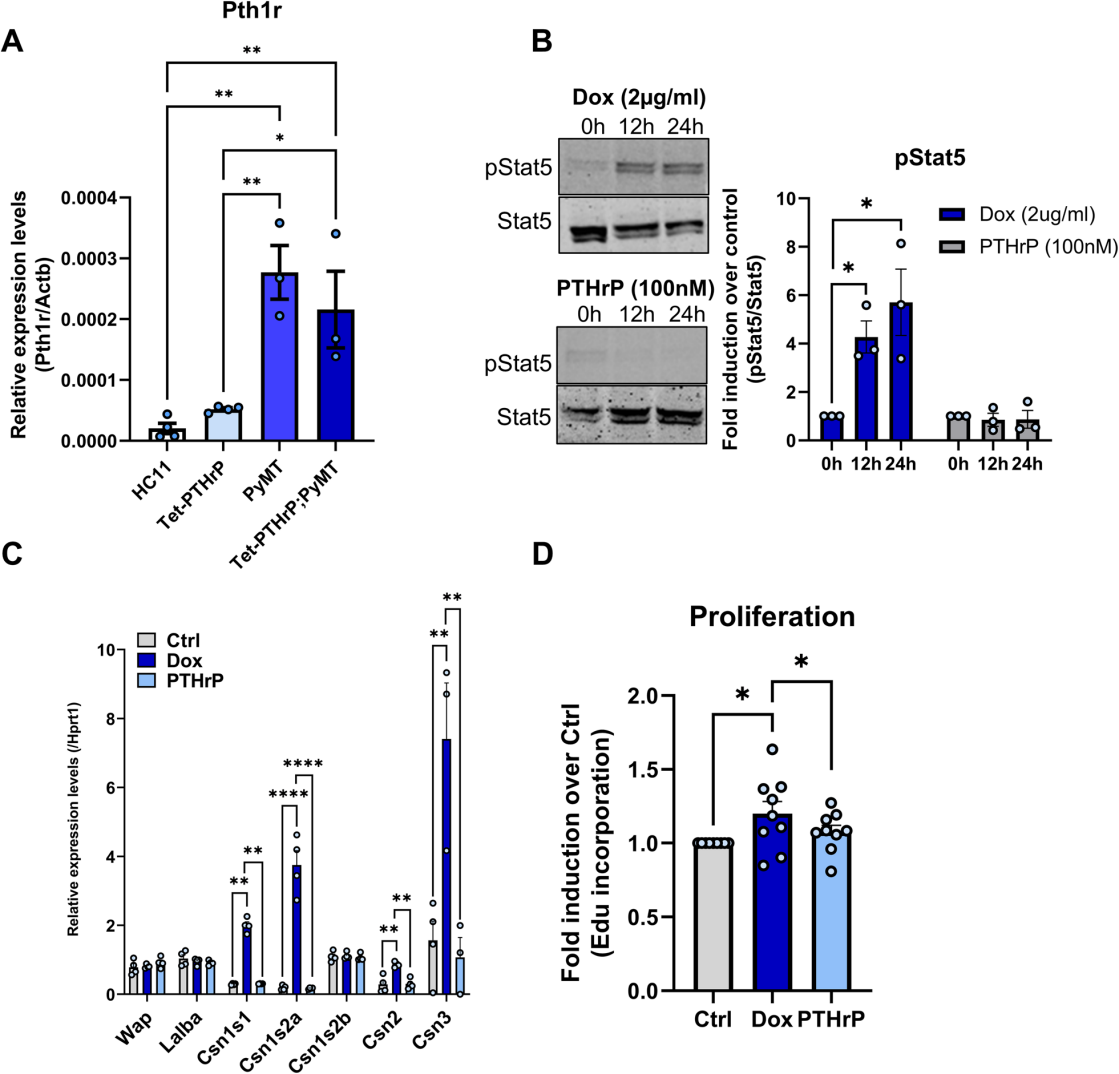
Overexpression of PTHrP, but not exogenously added PTHrP, activates Stat5 in tumor cells. **A** QPCR analysis of PTH1R expression in RNA from cultured HC11, Tet-PTHrP, PyMT and Tet-PTHrP;PyMT cells. *Actb* was used as housekeeping gene. A minimum of *n* = 3 per group. Tet-PTHrP;PyMT tumor cells were treated with Dox (2 µg/ml) or PTHrP 1–34 (100 nM) and protein lysates and RNA was prepared. **B** Western blot analysis of protein lysates. Left, representative immunoblots of p(Tyr694)Stat5 and total Stat5 are shown. Right, densitometric quantification of the western blots. *N* = 3. **C** QPCR analysis of the indicated milk proteins. *Hprt1* was used as housekeeping gene. *N* = 3. **D** Edu incorporation in cultured Tet-PTHrP;PyMT tumor cells in response to Dox or PTHrP treatment versus control. *N* = 9. Bars represent mean ± SEM, ****p < 0.0001, **p < 0.01, *p < 0.05

PTHrP can signal through autocrine/paracrine mechanisms involving the activation of its cell surface receptor (PTH1R) or, alternatively, through intracrine/nuclear mechanisms [3, 4, 6]. Tran and colleagues had previously described correlations between immunostaining for nuclear PTHrP and nuclear pSTAT5 expression in human breast tumors [10]. Therefore, we hypothesized that PTHrP triggered secretory differentiation in breast cancer cells through an intracrine pathway involving Stat5 activation. In order to test this idea, we isolated cells from mammary tumors taken from Tet-PTHrP;PyMT mice and first confirmed that they expressed the PTH1R. Consistent with prior literature [28, 73, 74], we found low levels of *Pth1r* expression in non-transformed HC11 mouse mammary epithelial cells or in the MECs derived from Tet-PTHrP mice, but found increased levels of *Pth1r* expressed in PyMT tumor cells derived from either MMTV-PyMT mice or from Tet-PTHrP;PyMT mice (Fig. 8A). Next, we treated the isolated tumor cells with vehicle, with Dox to induce endogenous PTHrP expression, or with exogenous PTHrP (100 nM) added to the media. Treating the cells with Dox caused increased pStat5 levels as assessed by Western analysis, whereas adding PTHrP to the media of the cells did not (Fig. 8B). In both circumstances, cells were cultured in the absence of prolactin. Similarly, Dox treatment was associated with an increase in the expression of various milk proteins by QPCR including *Csn1s1*, *Csn1s2a*, *Csn2* and *Csn3*, which, again, was not reproduced by treatment with exogenous PTHrP (Fig. 8C). Finally, we examined cell proliferation as assessed by BrdU incorporation. Induction of PTHrP with Dox treatment led to an increase in proliferation of the cells while treatment with exogenous PTHrP did not (Fig. 8D). These results suggest that the effects of PTHrP are cell autonomous, independent of prolactin stimulation, and mediated by intracrine actions of PTHrP.

**Fig. 9:**
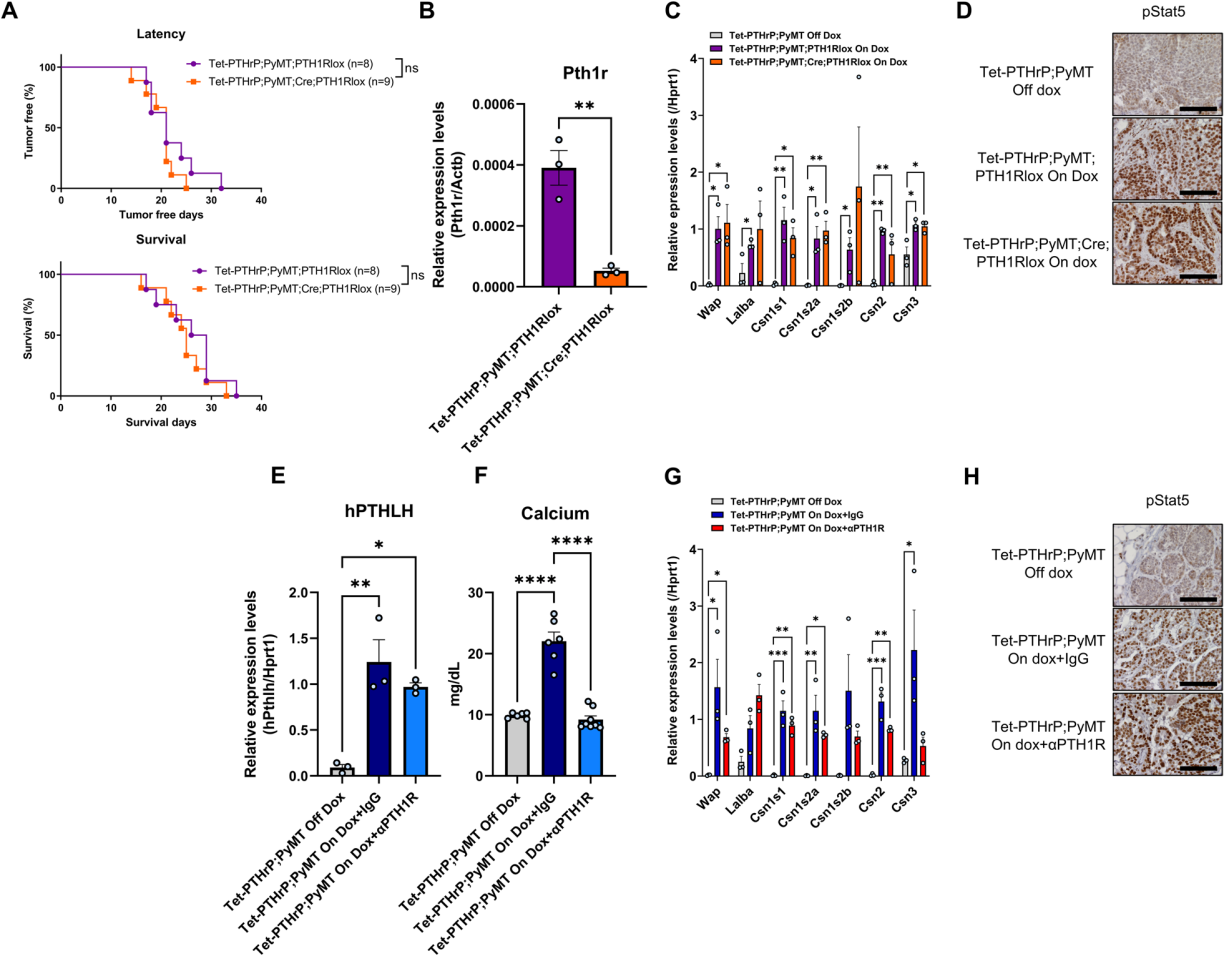
Knocking down or blocking PTH1R in tumors does not prevent the effects of PTHrP overexpression in vivo. **A** Kaplan–Meier analysis of tumor onset (top) and survival (bottom) of Tet-PTHrP;PyMT;PTH1RLox and Tet-PTHrP;PyMT;Cre;PTH1RLox mice treated with Dox from birth. **B** Relative expression of PTH1R in RNA from isolated tumor cells. *N* = 3. **C**, **G** QPCR analysis of indicated genes. *Hprt1* was used as housekeeping gene. *N* = 3. **D**, **H** Representative immunohistochemical staining for nuclear pStat5 in tumor sections from Tet-PTHrP;PyMT;Cre;PTH1RLox mice treated with Dox and controls and from Tet-PTHrP;PyMT treated with Dox and an anti-PTH1R antibody (αPTH1R) and controls. *N* = 3, Scale bar 100 µm. **E** QPCR analysis of hPTHLH expression in RNA from whole tumors. *Hprt1* was used as housekeeping gene. *N* = 3. **F** Serum calcium concentration. A minimum *n* = 6. Bars represent mean ± SEM, ****p < 0.0001, ***p < 0.001, **p < 0.01, *p < 0.05

We next tested whether the effects of PTHrP on tumor cell growth and differentiation in vivo depended on signaling through the PTH1R by engineering mice with MMTV-Cre-mediated disruption of the *Pth1r* gene in the setting of tetracycline-regulated PTHrP overexpression and MMTV-PyMT-mediated mammary tumorigenesis. MMTV-rtTA;TetO-PTHrP; MMTV-PyMT; MMTV-Cre;PTH1R^lox/lox^ (Tet-PTHrP;PyMT;Cre;PTH1RLox) mice on Dox were followed for the development of mammary tumors and compared to Tet-PTHrP;PyMT;PTH1RLox mice that lacked the Cre transgene. As shown in Fig. 9A, the incidence and latency of tumors as well as survival in Tet-PTHrP;PyMT;Cre;PTH1RLox mice was no different than in Tet-PTHrP;PyMT;PTH1RLox mice. Tumor cells isolated from these mice demonstrated successful reduction of *Pth1r* mRNA levels (Fig. 9B), but lack of PTH1R expression had no effect on the expression of typical markers of lactation such as *Wap*, *Lalba* and multiple casein genes in whole tumor lysates (Fig. 9C). In addition, ablation of the PTH1R had no effect on the expression of pSTAT5 in the nuclei of tumor cells (Fig. 9D). We further confirmed that the effects of PTHrP were independent of the PTH1R by treating Tet-PTHrP;PyMT mice with a blocking antibody against the PTH1R (anti-PTH1R) or IgG control at the same time Dox was provided (Fig. 9E–H). As expected, Tet-PTHrP;PyMT mice on Dox and treated with IgG developed hypercalcemia. However, Tet-PTHrP;PyMT mice on Dox and treated with anti-PTH1R antibody had normal calcium levels despite persistently elevated PTHrP levels, indicating that this treatment is highly effective in blocking systemic PTH1R signaling (Fig. 9E,F). In contrast, treatment with anti-PTH1R antibody did not prevent the induction of milk protein gene expression or STAT5 activation in tumor cells, demonstrating that the PTH1R is not required for PTHrP to trigger secretory differentiation in tumors (Fig. 9G,H). These results are consistent with the experiments in vitro suggesting that PTHrP acts in an intracrine manner.

### PTHLH gene expression in human breast cancer cells correlates with increased expression of genes associated with secretory differentiation

**Fig. 10:**
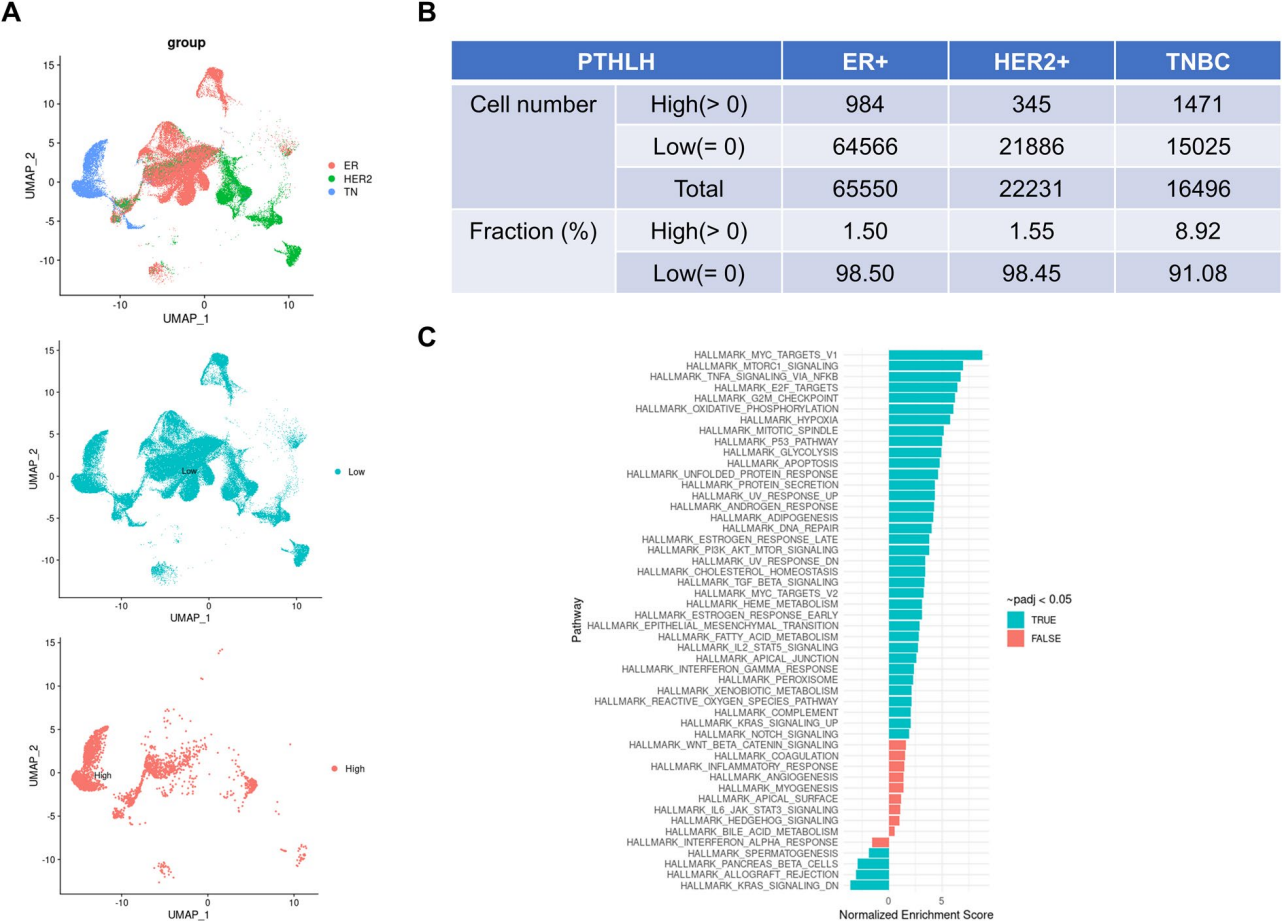
scRNA-seq analysis of PTHLH expression in human breast tumors. **A** Top panel: UMAP of clusters identified by scRNAseq of epithelial cells only (EpCAM +) separated by cancer subtype (ER + epithelial cells (*n* = 65,550), HER2 + epithelial cells (*n* = 22,231), and TNBC epithelial cells (*n* = 16,496)). Middle and Bottom Panel: UMAP overlays of PTHLH low and high expressing cells. **B** Table containing the proportion of PTHLH expressing cells in each cancer subtype. **C** fGSEA pathway analysis on DEGs from PTHLH-high versus PTHLH-low cells using pooled data from all tumor sub-types

In order to determine whether PTHrP production correlated with activation of STAT5-dependent, secretory differentiation pathways in human breast cancer, we examined recently published, single-cell sequencing data derived from 27 different human breast tumors (8 TNBC, 6 HER2-pos, 13 ER-pos) [54]. We were able to define *PTHLH-*high and *PTHLH-*low subsets from the pooled sequencing data of 86,277 individual epithelial tumor cells (Fig. 10A). The *PTHLH*-high cells were a distinct minority of the total cells and could be found at low levels in the three tumor sub-types. However, TNBCs had significantly more *PTHLH*-high cells (8.92%) than either HER2-positive (1.55%) or ER-positive (1.5%) tumors (Fig. 10B). We then defined the DEGs in *PTHLH-*high vs. *PTHLH*-low cells using pooled data from all tumor sub-types and performed functional pathways analyses using GSEA. We found that the DEGs were enriched in several pathways known to regulate aspects of lactation and milk production including protein secretion, fatty acid metabolism, PI3K-AKT-MTOR signaling and cholesterol homeostasis, as well as mitotic cell cycle and cell division processes (Fig. 10C). Importantly, GSEA confirmed that DEGs in *PTHLH*-high vs. *PTHLH*-low cells were significantly enriched in the hallmark IL-2-STAT5 signaling pathway [75]. Overall, these results suggest that the expression of PTHrP in human breast cancer cells is associated with the expression of genes involved in milk production and STAT5 signaling.

## Discussion

The data presented in this study demonstrate that overexpression of PTHrP in mammary epithelial cells activates a program of secretory differentiation. When treated with Dox to induce human PTHrP(1–141) expression, the mammary glands of virgin, Tet-PTHrP mice develop alveolar hyperplasia, express histological markers of secretory differentiation, activate milk protein gene expression, and accumulate intracellular lipids. These secretory changes are accompanied by the phosphorylation of STAT5 and an increase in the expression of Elf5, two pioneering transcription factors well described to cooperate in driving gene expression necessary for milk production [34–37, 60]. Consistent with the activation of these transcription factors, we found that PTHrP upregulates patterns of gene expression previously associated with lactation. It is also possible that PTHrP increases the pool of alveolar progenitors available for secretory differentiation rather than directly causing differentiation of the cells. However, alveolar hyperplasia and the expression of secretory differentiation markers are significantly reversed in response to the withdrawal of Dox, suggesting that they depend on the continuing presence of PTHrP. Previous results from our lab demonstrated that, although PTHrP expression is normally activated during lactation, mammary gland specific ablation of PTHrP affects systemic calcium metabolism during lactation but does not interfere with alveolar development or with overall milk secretion [16, 45]. Given the importance of lactation to mammalian reproduction, it is not surprising that the pathways leading to secretory differentiation would be redundant. Nevertheless, these new data clearly demonstrate that PTHrP is sufficient to induce secretory differentiation in luminal epithelial cells in the absence of pregnancy.

PTHrP overexpression also drives secretory differentiation in tumor cells in the MMTV-PyMT model of breast cancer. Tet-PTHrP;PyMT mice continuously exposed to Dox develop tumors in all mammary glands by 3–4 weeks of age, a dramatic decrease in tumor latency in comparison to Tet-PTHrP;PyMT mice not treated with Dox. While PTHrP promoted premature growth of PyMT-associated mammary tumors, overexpression of PTHrP alone did not efficiently induce tumors. Therefore, in this setting, PTHrP appeared primarily to promote tumor growth rather than initiate transformation. The decrease in tumor latency was accompanied by increased rates of proliferation in the tumors. As noted previously in vascular smooth muscle cells and in human and murine breast tumor cells, increased proliferation was associated with decreased expression of the cell cycle inhibitor, p27Kip1 [30, 76]. This acceleration of tumor development is consistent with the reciprocal results of Li et al., who showed that ablation of PTHrP expression in MMTV-PyMT tumors slowed tumor growth and was associated with reduced proliferation and increased apoptosis [31]. They are also consistent with prior results from our group demonstrating that ablation of the CaSR in MMTV-PyMT tumors or in human BT474 and MDA.MB-231.1833 cells reduced PTHrP expression, which was associated with a reduction in proliferation and increased expression of p27kip1 [30]. Importantly, these effects of PTHrP do not appear to result from an increase in PyMT expression although we cannot exclude the possibility that nuclear PTHrP affected the direct biological effects of PyMT itself. Thus, although there have been variable reports on the effects of PTHrP on human breast cancer cell lines, in mice, PTHrP clearly promotes proliferation in mammary tumor cells expressing PyMT.

While PTHrP accelerates the growth of PyMT tumors, it also promotes secretory differentiation. This is associated with an increase in the expression of differentiation markers, milk protein genes, Elf5, and pSTAT5. Despite an apparent shift to a more differentiated state, tumors continued to metastasize, and cells derived from the tumors overexpressing PTHrP were able to form new tumors when transplanted into non-transgenic mice. The histological appearance of the tumors in Tet-PTHrP;PyMT mice, their expression of pSTAT5 and the accumulation of milk-like secretions is reminiscent of a rare type of human breast cancer known as “secretory carcinoma of the breast” [77–81]. The majority of these tumors have been shown to contain t(12;15)(p13;q25) chromosomal translocations that results in a fusion oncogene (ETV6-NTRK3) consisting of the oligomerization domain of ETV6 fused to the protein tyrosine kinase domain of the neurotropin 3 receptor (NTRK3). Although most secretory carcinomas behave in an indolent manner, some patients develop metastatic lesions. When an ETV6-NTRK3 construct was knocked into mice, they developed mammary alveolar hyperplasia, followed by the development of multifocal tumors with short latency, again reminiscent of the effects of overexpressing PTHrP on PyMT-mediated tumorigenesis [80]. Although there is no known link between PTHrP expression and the expression or activity of NTRK3 or other neurotropin receptors, it has been suggested that the transforming ability of the ETV6-NTRK3 fusion oncogene relies on activation of the AP1 transcription complex [80]. Given the similarities between PTHrP overexpression in PyMT tumors and this model of secretory carcinomas, as well as the fact that PTHrP has been shown to activate AP1 signaling by increasing c-fos and/or JunB expression in several cell types, further study of potential interactions between PTHrP and AP1 signaling in breast cancer may be revealing [82, 83].

Multiple lines of evidence suggest that the effects of PTHrP on activating secretory differentiation pathways as well as on promoting tumor cell proliferation are mediated by an intracrine pathway rather than through its cell surface receptor. First, previous experiments overexpressing PTHrP in mammary gland myoepithelial cells did not lead to alveolar hyperplasia and secretory differentiation although, similar to the results reported here, it did inhibit ductal elongation during puberty [39, 73]. These differences are not compatible with a typical paracrine mode of action given that the 2 cell types overexpressing PTHrP in these different models are adjacent to each other. Instead, the different phenotypes in these models suggest that a cell-autonomous and cell-restricted mechanism of action drives the secretory differentiation. Second, in cells derived from Tet-PTHrP;PyMT mammary tumors, inducing PTHrP expression by treating them with Dox stimulates cell proliferation, activates STAT5 and increases milk protein gene expression, but treating the cells with exogenous PTHrP does not. Thus, PTHrP is sufficient to induce secretory differentiation, but only if produced within the tumor cells, suggesting a cell autonomous and intracrine mechanism. Third, reducing PTH1R expression in tumor cells does not alter tumor growth or secretory differentiation of the tumor cells, demonstrating that tumor expression of the PTH1R is not required for the observed phenotype. Lastly, treating tumor-bearing Tet-PTHrP;PyMT mice with anti-PTH1R antibodies corrects hypercalcemia but does not reverse STAT5 activation or reduce the expression of secretory markers, demonstrating that secreted PTHrP does not act systemically or on non-tumor cells in the microenvironment to induce paracrine cascades supporting secretory differentiation. These results are consistent with the observations of Tran et al., who previously reported that nuclear PTHrP staining correlates with nuclear pSTAT5 staining in human breast cancers [10]. In addition, Johnson et al. showed that PTHrP overexpression in MCF7 cells results in the downregulation of several pro-dormancy genes and suggested that these actions may occur through PTH1R-independent actions [74]. Finally, prior results from our laboratory have demonstrated that intracrine/nuclear actions of PTHrP downstream of the calcium-sensing receptor are important in modulating cell proliferation and survival in human breast cancer cell lines and in PyMT-induced mouse mammary tumors [30].

PTHrP is widely recognized to be important for the progression of osteolytic bone metastases from breast cancer [20, 84], although its role in the initiation, growth or progression of primary breast tumors is less clear. The results we report here agree with those of Li et al., demonstrating that PTHrP stimulates mammary tumor progression and results in shorter survival in MMTV-PyMT mice [31]. As compared to studies in mice, PTHrP has been variably suggested to either promote or to inhibit breast cancer cell proliferation, differentiation and death in human breast cancer cell lines [3, 5, 10, 30, 33]. Likewise, studies examining PTHrP staining in human breast cancers have reported differing correlations between PTHrP and tumor behavior. Some studies have reported that PTHrP expression correlates with estrogen receptor and progesterone receptor expression, a more differentiated histology, fewer metastases and a better prognosis [10, 26]. In contrast, other studies have suggested that increased PTHrP expression predicts worse survival and increases brain or bone metastases when measured in all breast tumors, in triple-negative breast cancers or in circulating tumor cells [25, 28, 85, 86]. One possible explanation for these conflicting results may be related to differing effects of PTHrP in luminal vs. triple negative sub-types of breast cancer. Another may relate to our observation that PTHrP overexpression results in the upregulation of STAT5 activation. STAT5 is critical to the proliferation and secretory differentiation of normal breast epithelial cells during pregnancy and lactation, but it seems to mirror PTHrP in having different effects on tumor progression in mice and humans. Loss of STAT5 impedes the development of tumors in T-antigen-dependent mouse models, while overexpression of wild-type or constitutively active STAT5 accelerates tumor formation in these models [36, 87–89]. By contrast, the activation of STAT5 in human breast cancers has generally been observed to be an indicator of more differentiated tumors and a better prognosis [10, 87, 90]. Our findings and those of Tran et al. mirror the previous literature in that PTHrP expression increases STAT5 and tumor progression in MMTV-PyMT mice, but PTHrP expression correlates with nuclear STAT5 expression and better outcome in human breast cancer. This may not be the entire bottom line given the recent report from Assaker and colleagues suggesting that tumor PTHrP expression at the time of diagnosis correlated with subsequent brain metastases and poor survival in patients with triple negative breast cancer (TNBC) [85]. Interestingly, we found the highest numbers of cells with elevated PTHrP gene expression in TNBC’s using single cell sequencing data. Furthermore, genes potentially involved in Stat5 signaling were enriched in TNBC cells expressing higher levels of PTHrP. Therefore, it is possible that interactions between PTHrP and STAT5 may have different consequences depending on the sub-type of breast cancer. Sorting out the details of when and how PTHrP affects different breast cancers in different fashions will be critical to understanding the reported association between the *PTHLH* gene and breast cancer in GWAS studies [22–24].

## Conclusions

In summary, we report that PTHrP overexpression activates STAT5, increases Elf5 expression, and leads to increased proliferation and secretory differentiation of both normal mammary epithelial cells and mammary tumor cells in mice. This is the result of an intracrine pathway rather than a function of secreted PTHrP. In addition, the greatest proportion of tumor cells with elevated PTHrP expression was found in triple negative breast cancers, where higher PTHrP mRNA levels correlated with an enrichment of STAT5-related gene expression. Our data confirm previous findings that nuclear PTHrP localization correlates with STAT5 activation and increased differentiation in human breast cancers [10]. Therefore, further work on PTHrP and breast cancer should focus on understanding how intracrine PTHrP signaling interacts with STAT5 signaling to modulate programs of secretory differentiation. In addition, it will be helpful to examine whether PTHrP plays an important role downstream of the ETV6-NTRK3 oncogene in secretory carcinomas of the breast. We believe that these lines of investigation may help to resolve conflicting published data regarding the overall effects of PTHrP on tumor behavior and patient survival. Given that *PTHLH* has been defined as a breast cancer susceptibility gene in GWAS studies, it is important to clarify the molecular mechanisms by which PTHrP affects breast cancer cell behavior.

## Supplementary Information

**Additional file 1. Figure S1: **Overexpression of PTHrP causes milk production in mammary glands from virgin mice. A) EdU incorporation in sections of mammary glands from Tet-PTHrP mice On Dox at 5 weeks of age. Magenta, Edu; Blue, DAPI. Scale bar 100 µm. B) Picture of the number 4 inguinal mammary gland from virgin, 13-week-old Tet-PTHrP mouse on dox showing milk accumulation. C) Densitometric quantification of the western blots for the indicated milk proteins and secretory differentiation markers shown in Figure 5. Bars represent mean ± SEM, n=3 per group. D) Circulating levels of plasma PTHrP and serum Prl concentration. Bars represent mean ± SEM, a minimum of n=3. ns: not significant.****p<0.0001 ***p<0.001 **p<0.01 *p<0.05.

**Additional file 2. Figure S2: **Alveolar hyperplasia and the mature secretory phenotype require ongoing exposure to PTHrP. Immunohistochemical staining of mammary gland sections. Representative images of an n=3 per group are shown. Scale bar 100µm.

**Additional file 3. Figure S3: **PTHrP overexpression causes microscopic tumors in Tet-PTHrP;PyMT mice as early as 10 days of age. A) Whole-mount analysis of carmine-stained, inguinal mammary glands from 10 day-old, Tet-PTHrP;PyMT mice on dox. Representative images of two out of three mice. Scale bars 1mm (left), 100µm (right). B) QPCR analysis of *Pymt* mRNA expression in RNA from whole tumor and from isolated tumor cells. *Actb* was used as a housekeeping gene. Bars represent mean ± SEM, a minimum n=3, ns: not significant.

**Additional file 4. Figure S4: **PTHrP induces secretory differentiation in PyMT tumor cells, without reversing their transformed state. A) H&E staining of tumors from different mouse genotypes and Dox treatments as detailed on the left. Representative images from 3 different tumors and mice per group. Scale bar 100µm. B) Picture of the third and fourth mammary gland containing tumors from Tet-PTHrP;PyMT mouse on Dox showing milk accumulation. C) H&E staining of lung sections from Tet-PTHrP;PyMT mice on Dox. Black boxes highlight lung metastases. Representative images of metastasis from 3 different mice. Scale bar 100µm. D) Representative immunohistochemical staining for nuclear pStat5 and IgG control in lung sections from Tet-PTHrP;PyMT with Dox and control off Dox. N=3, Scale bar 100µm. E) Plasma PTHrP and serum calcium concentration from WT mice on Dox transplanted with isolated Tet-PTHrP;PyMT tumor cells. Bars represent mean ± SEM, n=6.

**Additional file 5. Figure S5: **Global mRNA profiling in tumors of Tet-PTHrP;PyMT and PyMT mice on Dox. A) Volcano plot shows the log_2_ fold change and variance for all transcripts in PTHrP-overexpressing tumors relative to controls. Lines illustrate 2-fold changes and a padj of 0.01. Differentially expressed transcripts are highlighted in light blue and the number of genes increased or decreased is indicated. B) Pathway analysis on differentially expressed genes. Node size represents gene count; node color represents padj. C) Heatmap of STAT5-dependent mammary gland genes comparing Tet-PTHrP;PyMT vs PyMT mice on Dox using GSEA. N=3.

**Additional file 6. **Uncropped blot images from Fig. 2.

**Additional file 7. **Uncropped blot images from Fig. 6.

**Additional file 8.** Uncropped blot images from Fig. 8.

## Data Availability

The dataset supporting the conclusions of this article is available in the ArrayExpress database (http://www.ebi.ac.uk/arrayexpress) under accession number E-MTAB-11281.

## Abbreviations

PTHrP: Parathyroid hormone-related protein
PTH: Parathyroid hormone
PTH1R: PTH/PTHrP receptor 1
MMTV: Mouse mammary tumor virus long terminal repeat
ELF5: E74-like factor-5
TEB: Terminal end bud
MEC: Mammary epithelial cell
NKCC1: Sodium–potassium-chloride co-transporter 1
NPT2b: Sodium-phosphate transporter 2b
WAP: Whey acidic protein
LALBA: Alpha lactalbumin
DOX: Doxycycline
NF1B: Nuclear factor 1B
GSEA: Gene set enrichment analysis
DEG: Differentially expressed gene
BrdU: Bromodeoxyuridine
EdU: 5-Ethynyl-2′-deoxyuridine
TNBC: Triple negative breast cancer
ER: Estrogen receptor
