# Supplementary figures and images for: PTHrP induces STAT5 activation, secretory differentiation and accelerates mammary tumor development
Source: Breast Cancer Res. 2022 Apr 19;24:30. doi: 10.1186/s13058-022-01523-1 (PMC9020078; doi:10.1186/s13058-022-01523-1)

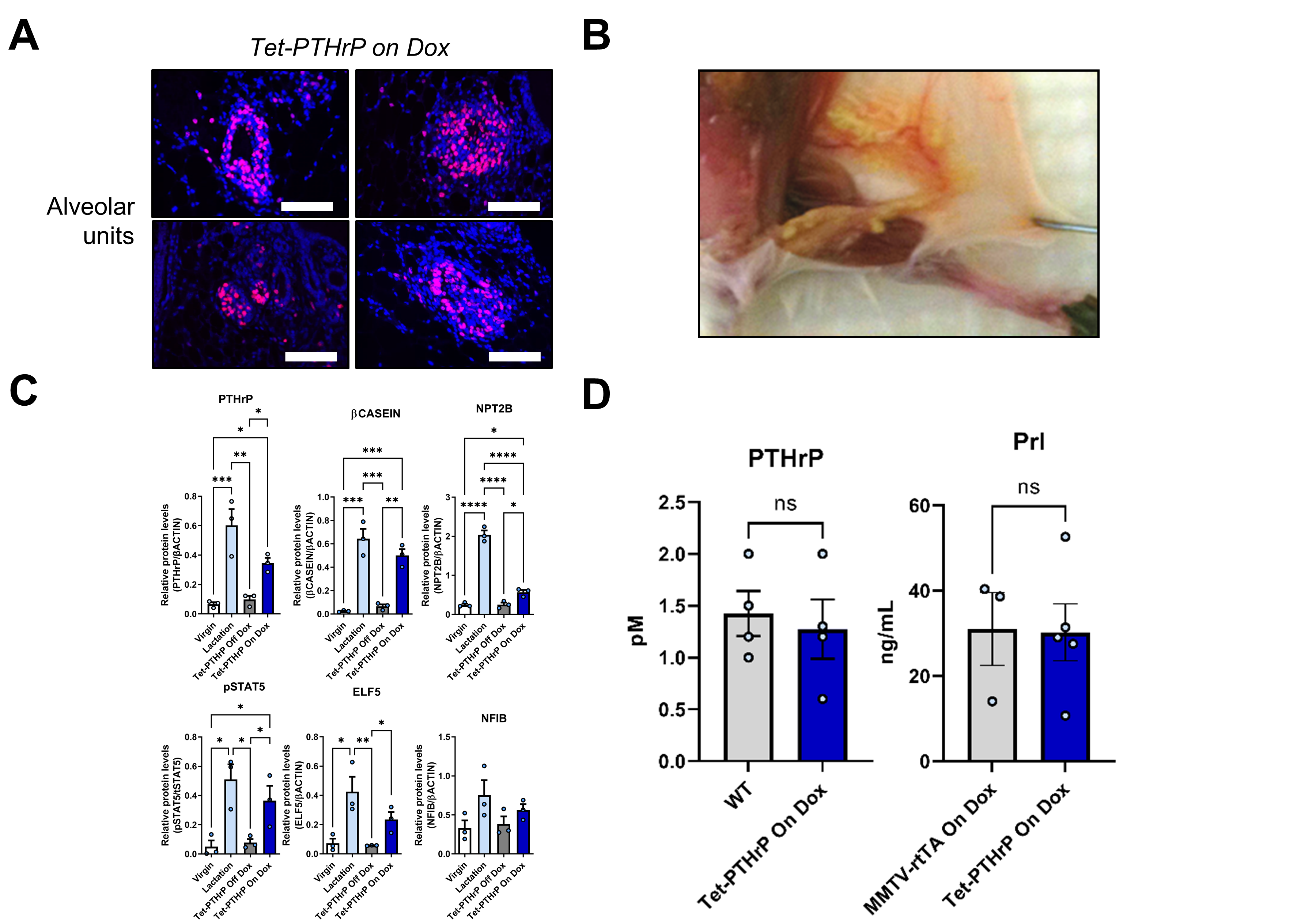

Supplement: Supplementary file 1 — Additional file 1. Figure S1: Overexpression of PTHrP causes milk production in mammary glands from virgin mice. A) EdU incorporation in sections of mammary glands from Tet-PTHrP mice On Dox at 5 weeks of age. Magenta, Edu; Blue, DAPI. Scale bar 100 µm. B) Picture of the number 4 inguinal mammary gland from virgin, 13-week-old Tet-PTHrP mouse on dox showing milk accumulation. C) Densitometric quantification of the western blots for the indicated milk proteins and secretory differentiation markers shown in Figure 5. Bars represent mean ± SEM, n=3 per group. D) Circulating levels of plasma PTHrP and serum Prl concentration. Bars represent mean ± SEM, a minimum of n=3. ns: not significant.****p<0.0001 ***p<0.001 **p<0.01 *p<0.05. [file 13058_2022_1523_MOESM1_ESM.tif]

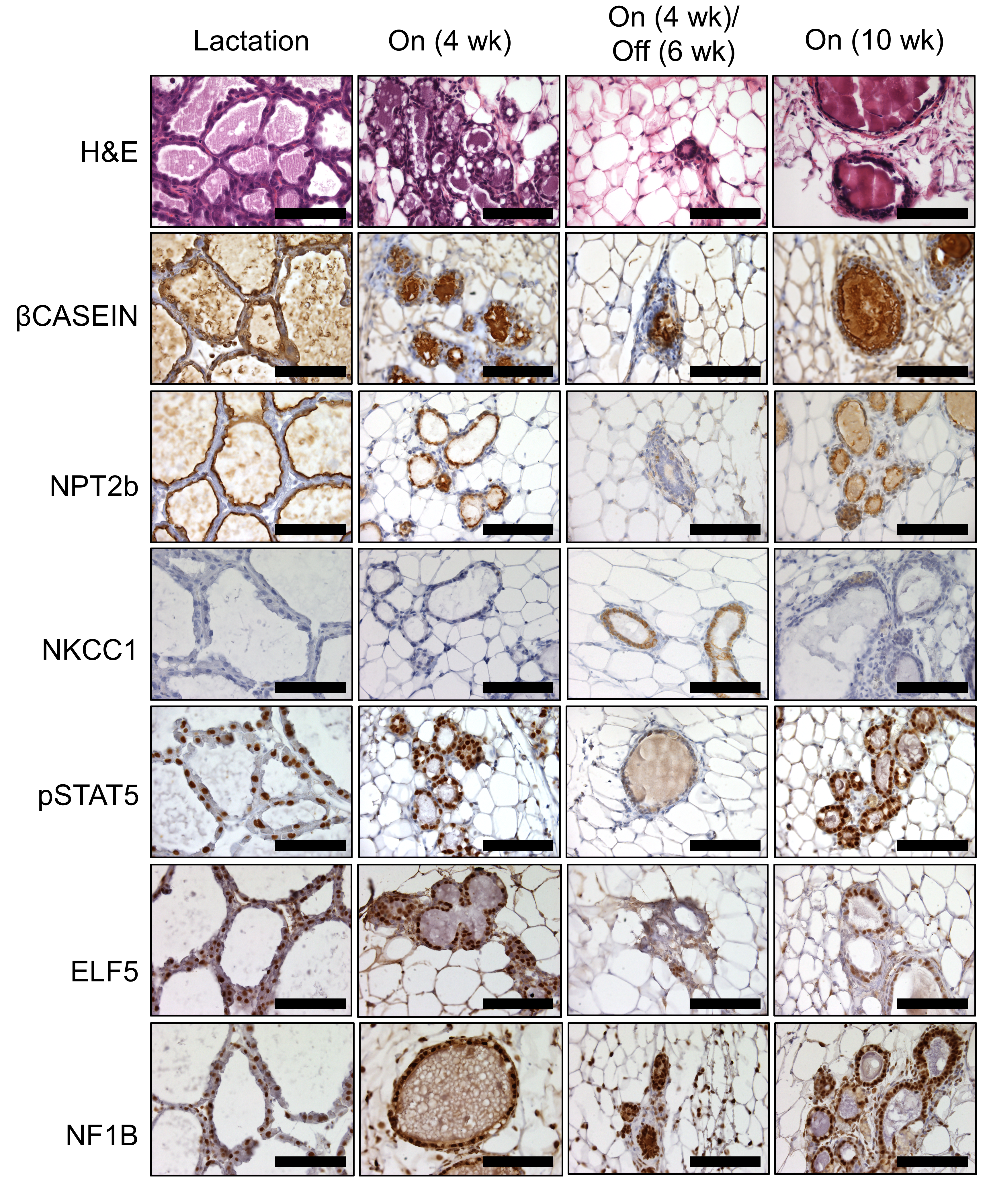

Supplement: Supplementary file 2 — Additional file 2. Figure S2: Alveolar hyperplasia and the mature secretory phenotype require ongoing exposure to PTHrP. Immunohistochemical staining of mammary gland sections. Representative images of an n=3 per group are shown. Scale bar 100µm. [file 13058_2022_1523_MOESM2_ESM.tif]

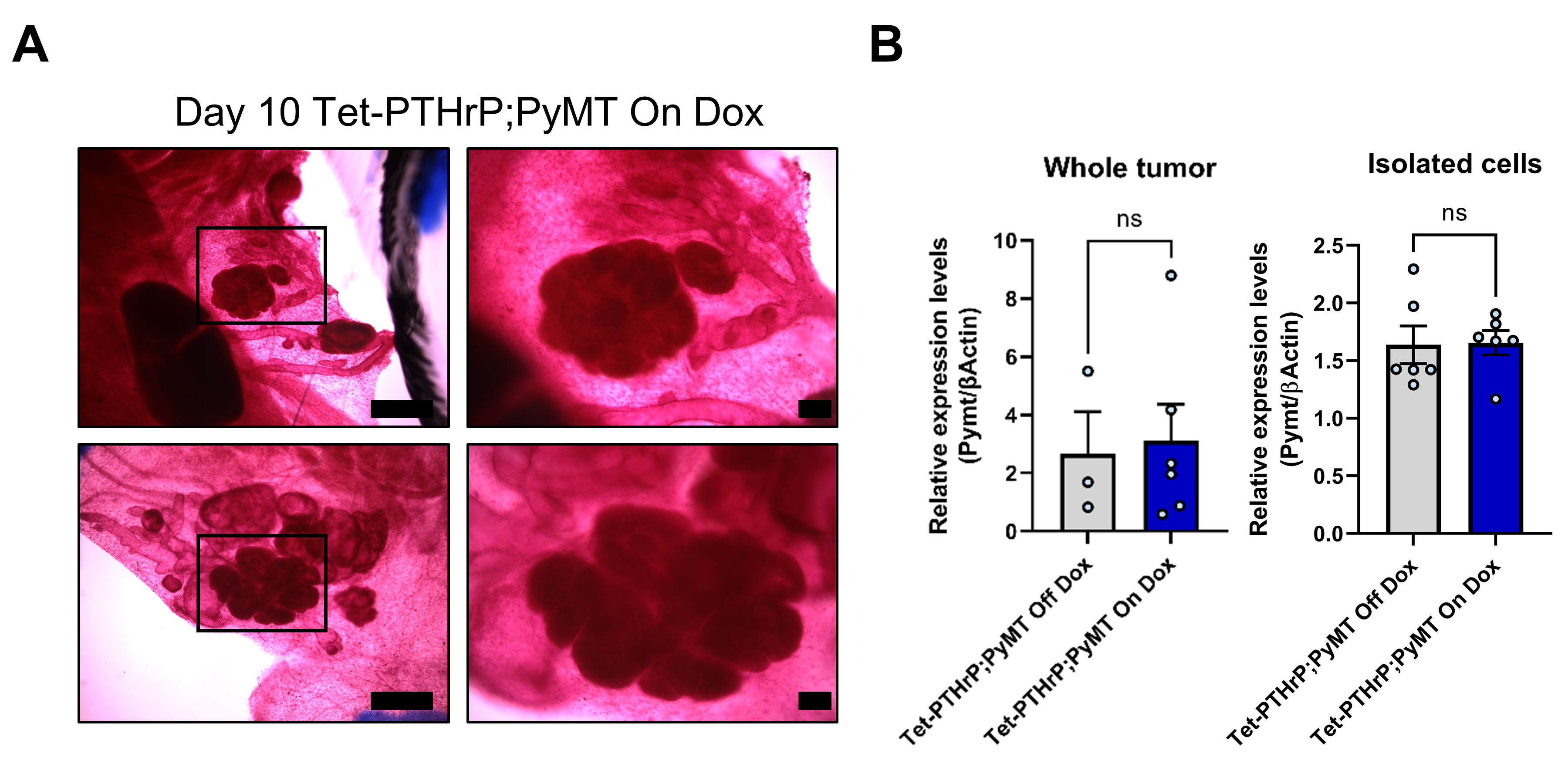

Supplement: Supplementary file 3 — Additional file 3. Figure S3: PTHrP overexpression causes microscopic tumors in Tet-PTHrP;PyMT mice as early as 10 days of age. A) Whole-mount analysis of carmine-stained, inguinal mammary glands from 10 day-old, Tet-PTHrP;PyMT mice on dox. Representative images of two out of three mice. Scale bars 1mm (left), 100µm (right). B) QPCR analysis of Pymt mRNA expression in RNA from whole tumor and from isolated tumor cells. Actb was used as a housekeeping gene. Bars represent mean ± SEM, a minimum n=3, ns: not significant. [file 13058_2022_1523_MOESM3_ESM.tif]

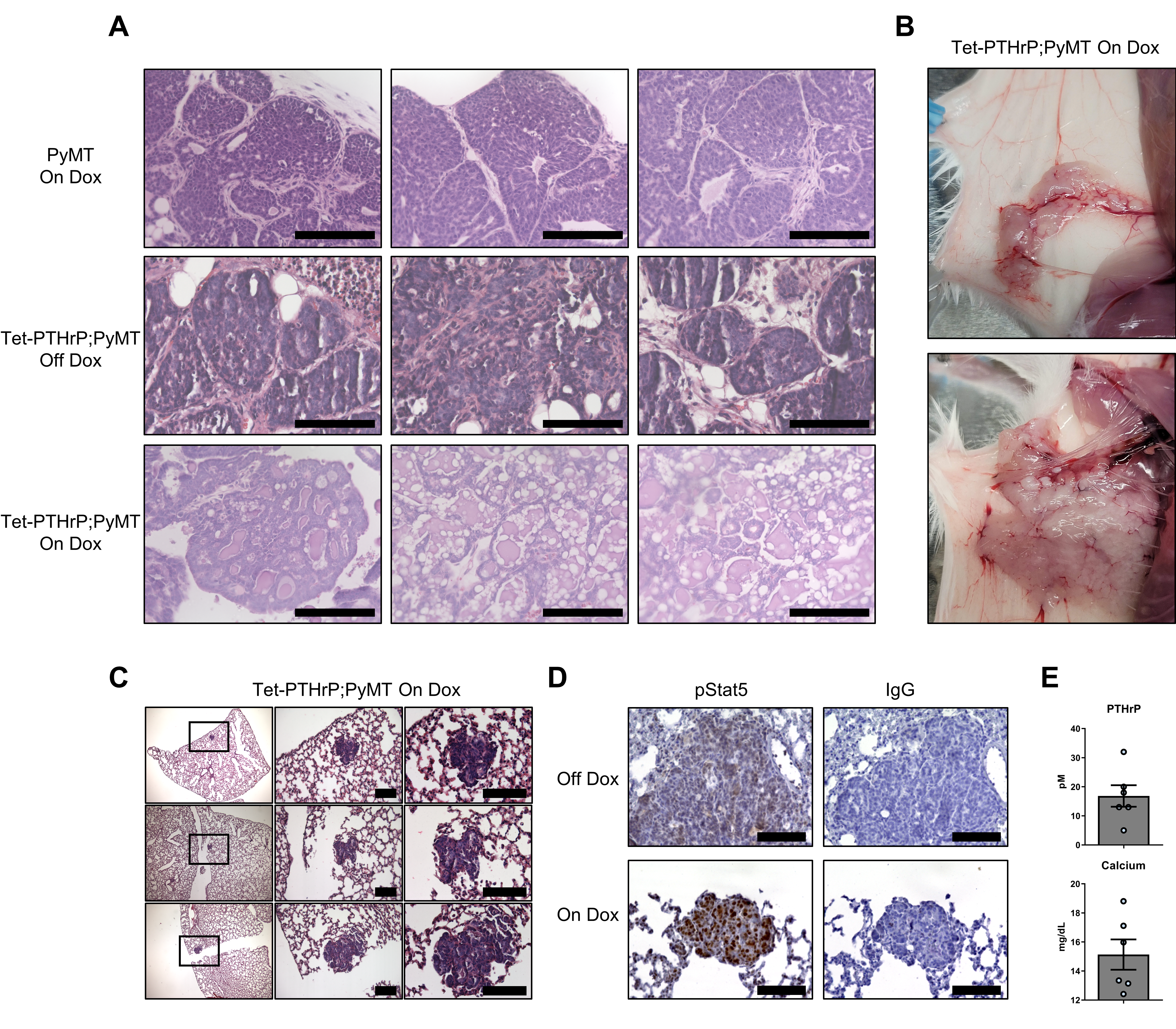

Supplement: Supplementary file 4 — Additional file 4. Figure S4: PTHrP induces secretory differentiation in PyMT tumor cells, without reversing their transformed state. A) H&E staining of tumors from different mouse genotypes and Dox treatments as detailed on the left. Representative images from 3 different tumors and mice per group. Scale bar 100µm. B) Picture of the third and fourth mammary gland containing tumors from Tet-PTHrP;PyMT mouse on Dox showing milk accumulation. C) H&E staining of lung sections from Tet-PTHrP;PyMT mice on Dox. Black boxes highlight lung metastases. Representative images of metastasis from 3 different mice. Scale bar 100µm. D) Representative immunohistochemical staining for nuclear pStat5 and IgG control in lung sections from Tet-PTHrP;PyMT with Dox and control off Dox. N=3, Scale bar 100µm. E) Plasma PTHrP and serum calcium concentration from WT mice on Dox transplanted with isolated Tet-PTHrP;PyMT tumor cells. Bars represent mean ± SEM, n=6. [file 13058_2022_1523_MOESM4_ESM.tif]

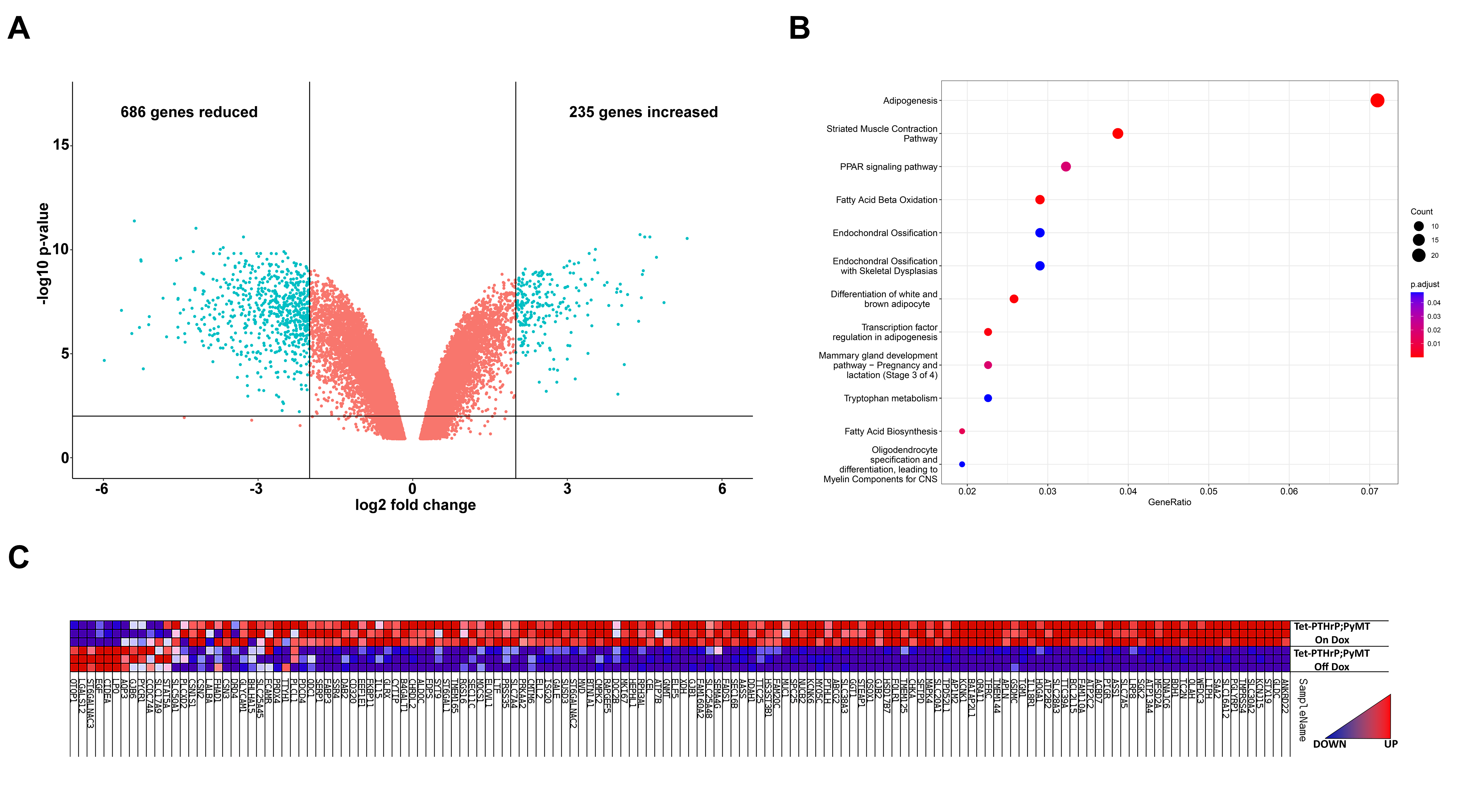

Supplement: Supplementary file 5 — Additional file 5. Figure S5: Global mRNA profiling in tumors of Tet-PTHrP;PyMT and PyMT mice on Dox. A) Volcano plot shows the log2 fold change and variance for all transcripts in PTHrP-overexpressing tumors relative to controls. Lines illustrate 2-fold changes and a padj of 0.01. Differentially expressed transcripts are highlighted in light blue and the number of genes increased or decreased is indicated. B) Pathway analysis on differentially expressed genes. Node size represents gene count; node color represents padj. C) Heatmap of STAT5-dependent mammary gland genes comparing Tet-PTHrP;PyMT vs PyMT mice on Dox using GSEA. N=3. [file 13058_2022_1523_MOESM5_ESM.tif]

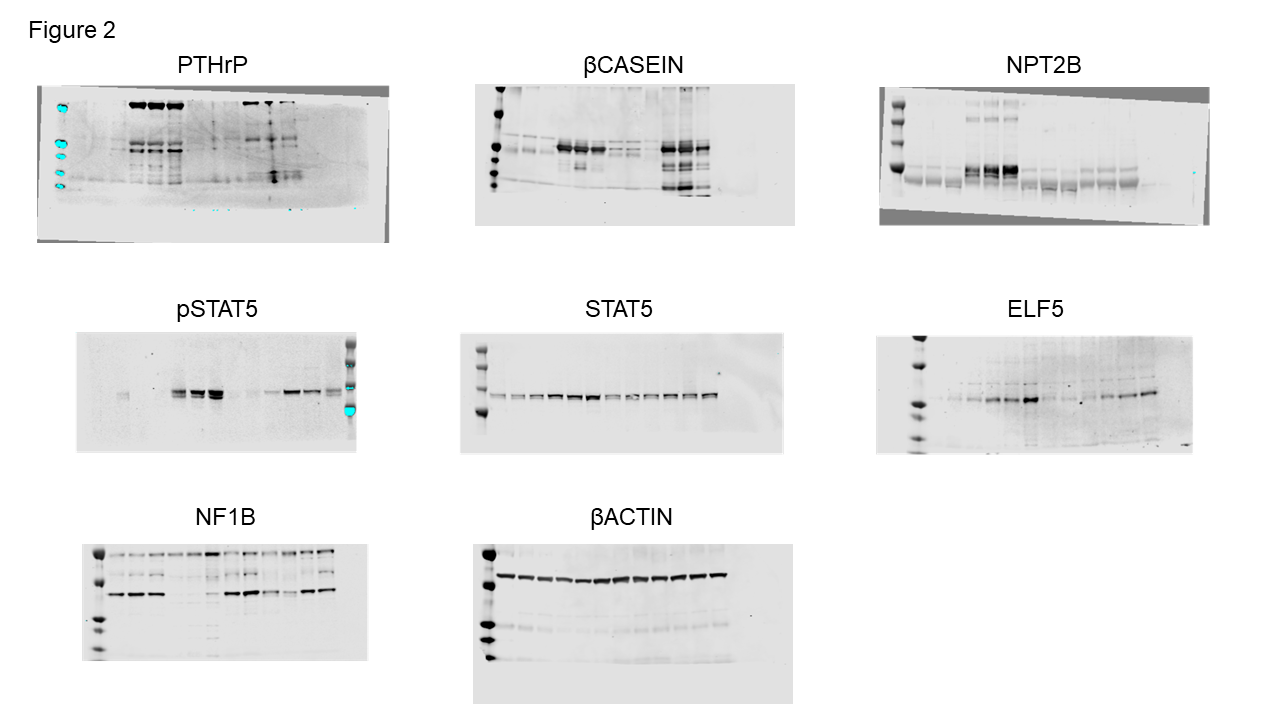

Supplement: Supplementary file 6 — Additional file 6. Uncropped blot images from Fig. 2. [file 13058_2022_1523_MOESM6_ESM.tif]

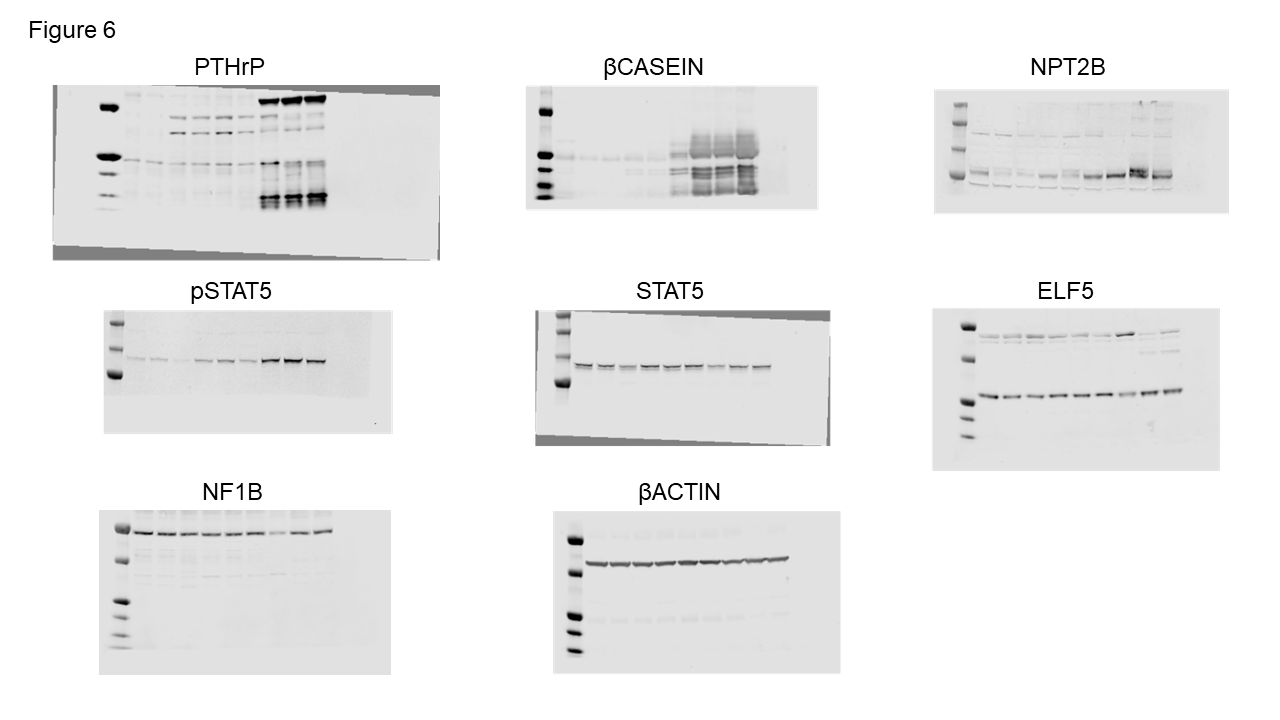

Supplement: Supplementary file 7 — Additional file 7. Uncropped blot images from Fig. 6. [file 13058_2022_1523_MOESM7_ESM.tif]

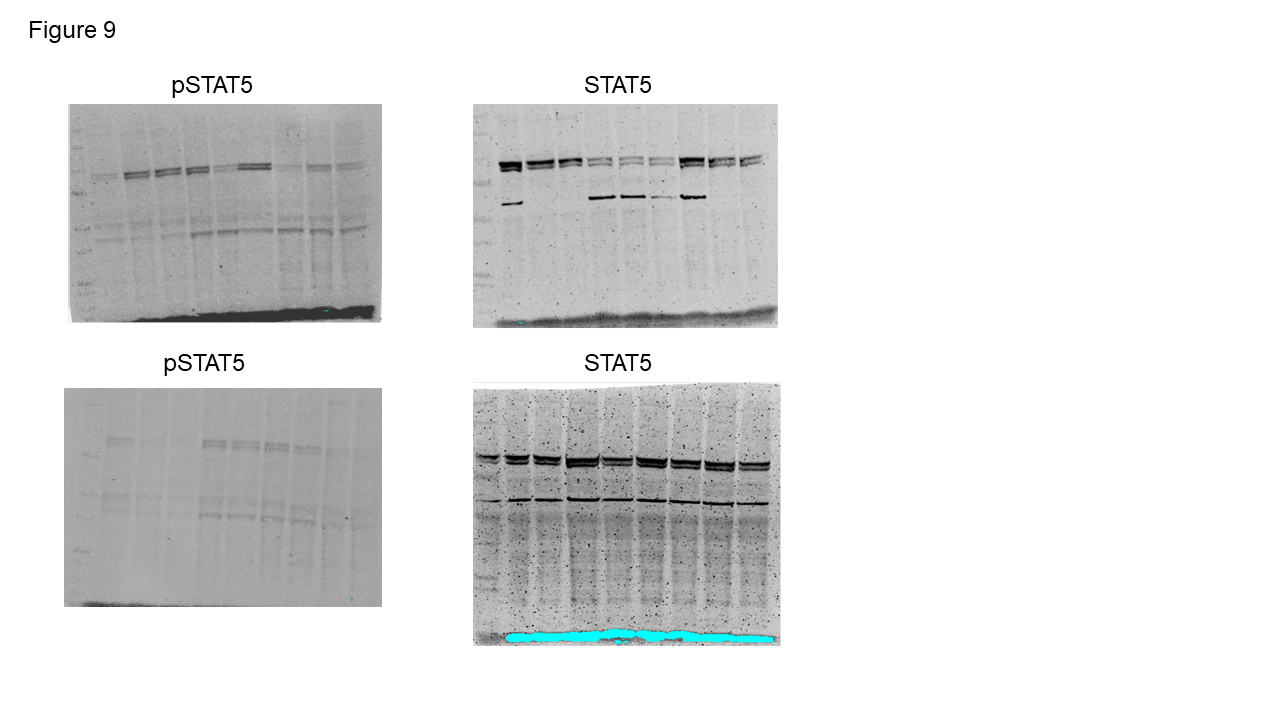

Supplement: Supplementary file 8 — Additional file 8. Uncropped blot images from Fig. 8. [file 13058_2022_1523_MOESM8_ESM.tif]
